# Long term cause specific mortality among 34 489 five year survivors of childhood cancer in Great Britain: population based cohort study

**DOI:** 10.1136/bmj.i4351

**Published:** 2016-09-01

**Authors:** Miranda M Fidler, Raoul C Reulen, David L Winter, Julie Kelly, Helen C Jenkinson, Rod Skinner, Clare Frobisher, Michael M Hawkins

**Affiliations:** 1 Centre for Childhood Cancer Survivor Studies, Institute of Applied Health Research, University of Birmingham, Birmingham B15 2TT, UK; 2 Department of Oncology, Birmingham Children’s Hospital, NHS Foundation Trust, Birmingham, UK; 3 Department of Paediatric and Adolescent Haematology and Oncology, and Children’s BMT Unit, Great North Children’s Hospital, Royal Victoria Infirmary, Newcastle upon Tyne, UK

## Abstract

**Objective** To determine whether modern treatments for cancer are associated with a net increased or decreased risk of death from neoplastic and non-neoplastic causes among survivors of childhood cancer.

**Design** Population based cohort study.

**Setting** British Childhood Cancer Survivor Study.

**Participants** Nationwide population based cohort of 34 489 five year survivors of childhood cancer with a diagnosis from 1940 to 2006 and followed up until 28 February 2014.

**Main outcome measures** Cause specific standardised mortality ratios and absolute excess risks are reported. Multivariable Poisson regression models were utilised to evaluate the simultaneous effect of risk factors. Likelihood ratio tests were used to test for heterogeneity or trend.

**Results** Overall, 4475 deaths were observed, which was 9.1 (95% confidence interval 8.9 to 9.4) times that expected in the general population, corresponding to 64.2 (95% confidence interval 62.1 to 66.3) excess deaths per 10 000 person years. The number of excess deaths from all causes declined among those treated more recently; those treated during 1990-2006 experienced 30% of the excess number of deaths experienced by those treated before 1970. The corresponding percentages for the decline in excess deaths from recurrence or progression and non-neoplastic causes were 30% and 60%, respectively. Among survivors aged 50-59 years, 41% and 22% of excess deaths were attributable to subsequent primary neoplasms and circulatory conditions, respectively, whereas the corresponding percentages among those aged 60 years or more were 31% and 37%.

**Conclusions** The net effects of changes in cancer treatments, and surveillance and management for late effects, over the period 1940 to 2006 was to reduce the excess number of deaths from both recurrence or progression and non-neoplastic causes among those treated more recently. Among survivors aged 60 years or more, the excess number of deaths from circulatory causes exceeds the excess number of deaths from subsequent primary neoplasms. The important message for the evidence based surveillance aimed at preventing excess mortality and morbidity in survivors aged 60 years or more is that circulatory disease overtakes subsequent primary neoplasms as the leading cause of excess mortality.

## Introduction

Long term survivors of childhood cancer remain at an increased risk of mortality when compared with that expected from the general population.^1^ ^2^ ^3^ ^4^ ^5^ ^6^ ^7^ Previous reports have shown that the principal cause of excess mortality in the short term is recurrence or progression of the initial cancer,^1^ ^2^ ^3^ ^8^ whereas subsequent primary neoplasms and non-neoplastic causes account for the majority of excess deaths long term.^1^ ^2^ ^5^

Treatment intensity has typically decreased more recently for children with a diagnosis of neoplasms with relatively good prognosis in order to prevent premature morbidity and mortality from treatment related side effects; conversely, treatment has intensified for neoplasms with poor prognoses in order to improve survival. Few studies have addressed late mortality in relation to treatment period,^3^ ^4^ ^7^ ^8^ ^9^ and those that have were restricted by narrow treatment time spans, insufficient person years at risk, and small numbers of deaths, which limited statistical power and inhibited detailed classification and investigation of cause specific deaths.

Thus, in this study we aimed to address these previous limitations by investigating the risk of late cause specific mortality after treatment across almost seven decades (1940-2006) within the recently extended British Childhood Cancer Survivor Study (BCCSS). The BCCSS now includes 34 489 five year survivors of childhood cancer, which is 6056, 7846, and 14 006 more five year survivors than that included in the Childhood Cancer Survivor Study (CCSS),^7^ Surveillance, Epidemiology and End Results Program (SEER),^8^ and Nordic countries,^3^ respectively. Our study also included 1496, 1662, and 2407 more deaths than that reported by these same studies, respectively. Owing to these strengths, we investigated the impact of factors related to treatment period on the risk of specific causes of death and the pattern of excess deaths among survivors aged more than 50 years.

## Methods

### British Childhood Cancer Survivor Study

The BCCSS is a population based cohort that comprises 34 489 five year survivors of childhood cancer with a diagnosis under the age of 15 years from 1940 to 2006 in Britain. The cohort was ascertained by using the National Registry of Childhood Tumours, which has an approximately 99% ascertainment rate.^10^ The study is maintained at the Centre of Childhood Cancer Survivor Studies and additional details on the study and its objectives can be found at www.bccss.bham.ac.uk. Ethical approval for the study was obtained from the National Research Ethics Service, and the Confidentiality Advisory Group consented to processing identified data without individual patient consent.

### Death ascertainment

To ascertain each survivor’s vital and emigration status, the entire BCCSS cohort was linked by the Health and Social Care Information Centre (England and Wales) and National Health Service Central Register (Scotland) to their NHS registration systems using the survivor’s NHS number, first name, middle initial, current surname, and date of birth; for survivors who were not electronically matched, manual matching was then undertaken through collaboration with the Health and Social Care Information Centre and NHS Central Register. This linkage provided the death certificate and underlying cause of death, as coded by the Office for National Statistics (England and Wales) and National Records of Scotland (Scotland), using the relevant *International Classification of Diseases* in use at date of death. We then classified the underlying cause of death, as coded on the death certificate, using the principal sections of the relevant revision of the *International Classification of Diseases*. However, as it was possible that a death from cancer could be due to a recurrence or progression of the original childhood cancer or a subsequent primary neoplasm, we (MMF, RCR, DLW, and MMH) reviewed all deaths due to neoplastic causes to determine the appropriate neoplastic specific cause of death (either recurrence or progression or subsequent primary neoplasm); death certificates, medical notes, and autopsy reports were utilised in the review and if uncertainty remained we attributed the cause of death to recurrence or progression. Follow-up for mortality commenced at the date of five year survival and continued until the first occurrence of emigration, death, or 28 February 2014.

### Statistical analyses

Using standard cohort techniques, we calculated standardised mortality ratios and absolute excess risks.^11^ The standardised mortality ratio was defined as the number of observed deaths divided by the expected number of deaths. The absolute excess risk was defined as the observed minus the expected number of deaths divided by person years at risk multiplied by 10 000. We calculated expected numbers by multiplying the person years for each sex, age (five year bands), and calendar year (one year bands) specific stratum by the corresponding mortality rate for the general population and then summing the expected numbers across the strata.^11^ ^12^ As a principal objective of this study was to assess trends in excess mortality with treatment period, we fitted univariable and multivariable Poisson regression models, where standardised mortality ratios and absolute excess risks were calculated from the univariate model and excess mortality ratios, which are essentially the ratio of absolute excess risks adjusting for other factors, were calculated from the multivariable model. We used the multivariable model to evaluate the simultaneous effect of several factors: sex, type of first primary neoplasm, age at cancer diagnosis, treatment period, attained age, and years of follow-up. Because of strong collinearity, we did not include attained age and years of follow-up in the same Poisson regression model. To test for heterogeneity or trend, we used likelihood ratio tests within Poisson regression models. All analyses were completed using Stata 13.1 statistical software,^13^ where the criterion for statistical significance was a two sided P<0.05.

### Patient involvement

Two patient representatives attend the BCCSS Steering Group meetings. Survivors overall showed their overwhelming support for the study by returning 10 488 questionnaires, representing 80% of those sent. Almost all of the survivors who completed the questionnaire requested to receive study newsletters, the means by which we inform them of the findings of the research.

## Results

### Study characteristics

The cohort was followed up for a total of 620 753 person years, with a median follow-up of 15.2 (range 0.0-68.7) years from five year survival and to a median attained age of 27.0 (range 5.5-85.6) years (table 1). When we assessed the cohort by follow-up, 31 582 person years were observed beyond 40 years; by attained age, 21 696 person years were observed beyond 50 years. Overall, 4475 people (13.0%) had died by the study exit date. Supplementary eTable 1 reports the distribution of the cohort characteristics by treatment period.

**Table 1 tbl1:** Cohort characteristics of the British Childhood Cancer Survivor Study

Characteristics	Dead (%)	Alive (%)	Total No
Overall	4475 (13.0)	30 014 (87.0)	34 489
Male	2629 (13.9)	16 310 (86.1)	18 939
Female	1846 (11.9)	13 704 (88.1)	15 550
First primary neoplasm type:			
CNS (excluding PNET)	1334 (19.1)	5636 (80.9)	6970
CNS PNET	340 (28.4)	858 (71.6)	1198
Leukaemia (excluding AML)	1104 (11.6)	8398 (88.4)	9493
AML	82 (8.4)	899 (91.6)	981
Hodgkin’s lymphoma	331 (14.8)	1903 (85.2)	2234
Non-Hodgkin’s lymphoma	131 (8.5)	1418 (91.5)	1549
Neuroblastoma	144 (9.4)	1391 (90.6)	1535
Non-heritable retinoblastoma	31 (3.1)	975 (96.9)	1006
Heritable retinoblastoma	138 (18.4)	612 (81.6)	750
Wilms’s tumour	184 (7.7)	2204 (92.3)	1388
Bone sarcoma	198 (16.6)	997 (83.4)	1195
Soft tissue sarcoma	253 (11.8)	1894 (88.2)	2147
Other	205 (6.7)	2838 (93.3)	3043
Age at diagnosis (years):			
0-4	1662 (10.6)	14 035 (89.4)	15 697
5-9	1351 (14.6)	7913 (85.4)	9264
10-14	1462 (15.3)	8066 (84.7)	9528
Treatment period:			
1940-69	1329 (35.5)	2417 (64.5)	3746
1970-79	1247 (23.2)	4132 (76.8)	5379
1980-89	942 (13.2)	6205 (86.8)	7147
1990-99	703 (7.0)	9328 (93.0)	10 031
2000-06	254 (3.1)	7932 (96.9)	8186
Years from diagnosis:			
Median (range)	6.1 (0.0-65.3)	16.2 (0.0-68.7)	15.2 (0.0-68.7)
5-9	2054 (37.9)	3368 (62.2)	5422
10-19	1060 (9.1)	10 561 (90.9)	11 621
20-29	548 (6.9)	7352 (93.1)	7900
30-39	417 (7.7)	5026 (92.3)	5443
40-49	277 (9.9)	2529 (90.1)	2806
50-59	103 (8.9)	1052 (91.1)	1155
≥60	16 (11.3)	126 (88.7)	142
Attained age (years) at exit:			
Median (range)	20.0 (5.5-79.8)	27.9 (5.8-85.6)	27.0 (5.5-85.6)
5-9	416 (48.9)	435 (51.1)	851
10-19	1831 (21.7)	6614 (78.3)	8445
20-29	1003 (9.4)	9730 (90.7)	10 733
30-39	535 (8.1)	6083 (91.9)	6618
40-49	364 (7.5)	4486 (92.5)	4850
50-59	238 (11.4)	1859 (88.7)	2097
≥60	88 (9.8)	807 (90.2)	895

CNS=central nervous system; PNET=primitive neuroectodermal tumour; AML=acute myeloid leukaemia.

### All causes of death

Overall, survivors experienced 9.1 times (95% confidence interval 8.9 to 9.4) more deaths than that expected from the general population, which corresponded to 64.2 (95% confidence interval 62.1 to 66.3) excess deaths per 10 000 person years (table 2). With regards to absolute excess risks, over 50 excess deaths per 10 000 person years were observed for survivors of central nervous system (CNS) neoplasms (excluding primitive neuroectodermal tumour), CNS primitive neuroectodermal tumour, leukaemia (excluding acute myeloid leukaemia), acute myeloid leukaemia, Hodgkin’s lymphoma, heritable retinoblastoma, bone sarcoma, and soft tissue sarcoma (table 3). When assessed by treatment period, the absolute excess risks decreased significantly (P for trend <0.01) among those with a more recent diagnosis. After adjusting for sex, type of first primary neoplasm, age at diagnosis, and attained age, those with a diagnosis of cancer from 1990 to 2006 experienced 30% (excess mortality ratio 0.3, 95% confidence interval 0.3 to 0.4) of the excess deaths observed among those with a diagnosis before 1970 (table 4).

**Table 2 tbl2:** Observed and expected deaths, standardised mortality ratio, and absolute excess risk of specific causes of death

Causes of death	Observed/expected	SMR (95% CI)	Absolute excess risk (95% CI)
All causes	4475/490.9	9.1 (8.9 to 9.4)	64.2 (62.1 to 66.3)
Recurrence or progression	2626/0.0	NA	42.3 (40.7 to 43.9)
Subsequent primary neoplasm	795/126.9	6.3 (5.8 to 6.7)	10.8 (9.9 to 11.7)
Non-neoplastic	1054/364.0	2.9 (2.7 to 3.1)	11.1 (10.1 to 12.1)
Circulatory	300/78.0	3.8 (3.4 to 4.3)	3.6 (3.0 to 4.1)
Respiratory	164/24.2	6.8 (5.8 to 7.9)	2.3 (1.8 to 2.7)
Nervous	98/23.0	4.3 (3.5 to 5.2)	1.2 (0.9 to 1.5)
Infection	67/9.1	7.4 (5.7 to 9.4)	0.9 (0.7 to 1.2)
Digestive	63/30.6	2.1 (1.6 to 2.6)	0.5 (0.3 to 0.8)
Perinatal	42/9.5	4.4 (3.2 to 6.0)	0.5 (0.3 to 0.7)
Endocrine	32/10.5	3.1 (2.1 to 4.3)	0.3 (0.2 to 0.5)
Genitourinary	30/3.3	9.2 (6.2 to 13.2)	0.4 (0.3 to 0.6)
Musculoskeletal	18/3.0	6.0 (3.5 to 9.4)	0.2 (0.1 to 0.4)
Mental	15/13.3	1.1 (0.6 to 1.9)	0.0 (−0.1 to 0.1)
Blood	16/2.1	7.5 (4.3 to 12.2)	0.2 (0.1 to 0.3)
External*	188/151.7	1.2 (1.1 to 1.4)	0.6 (0.2 to 1.0)
Other	21/5.7	3.7 (2.3 to 5.7)	0.2 (0.1 to 0.4)

SMR=standardised mortality ratio; NA=not applicable.

*Includes deaths due to transportation accidents, falls, drowning, fire, suicide, etc.

**Table 3 tbl3:** Standardised mortality ratios and absolute excess risks for deaths due to all causes, recurrence or progression, subsequent primary neoplasm, and non-neoplastic causes, by potential explanatory factors

Factors	All causes				Recurrence or progression			Subsequent primary neoplasms				Non-neoplastic causes		
	Observed/expected	SMR (95% CI)	AER (95% CI)		Observed/expected	AER (95% CI)		Observed/expected	SMR (95% CI)	AER (95% CI)		Observed/expected	SMR (95% CI)	AER (95% CI)
Overall	4475/490.9	9.1 (8.9 to 9.4)	64.2 (62.1 to 66.3)		2626/0.0	42.3 (40.7 to 43.9)		795/126.9	6.3 (5.8 to 6.7)	10.8 (9.9 to 11.7)		1054/364.0	2.9 (2.7 to 3.1)	11.1 (10.1 to 12.1)
Male	2629/332.7	7.9 (7.6 to 8.2)	68.2 (65.2 to 71.2)		1536/0.0	45.6 (43.3 to 47.9)		454/66.6	6.8 (6.2 to 7.5)	11.5 (10.3 to 12.7)		639/266.1	2.4 (2.2 to 2.6)	11.1 (9.6 to 12.5)
Female	1846/158.3	11.7 (11.1 to 12.2)	59.4 (56.5 to 62.4)		1090/0.0	38.4 (36.1 to 40.7)		341/60.3	5.7 (5.1 to 6.3)	9.9 (8.6 to 11.2)		415/97.9	4.2 (3.8 to 4.7)	11.2 (9.8 to 12.6)
P for heterogeneity*		<0.01	<0.01			<0.01			0.25	0.18			<0.01	0.83
First primary neoplasm type:														
CNS (excluding PNET)	1334/115.5	11.5 (10.9 to 12.2)	97.7 (91.9 to 103.4)		808/0.0	64.8 (60.3 to 69.2)		148/32.7	4.5 (3.8 to 5.3)	9.2 (7.3 to 11.2)		378/82.9	4.6 (4.1 to 5.0)	23.7 (20.6 to 26.7)
CNS PNET	340/14.6	23.3 (20.9 to 25.9)	174.1 (154.7 to 193.4)		215/0.0	115.0 (99.6 to 130.4)		72/3.3	21.6 (16.9 to 27.2)	36.7 (27.8 to 45.6)		53/11.3	4.7 (3.5 to 6.2)	22.3 (14.7 to 30.0)
Leukaemia (excluding AML)	1104/71.2	15.5 (14.6 to 16.5)	71.1 (66.6 to 75.6)		830/0.0	57.1 (53.3 to 61.0)		126/12.7	9.9 (8.3 to 11.8)	7.8 (6.3 to 9.3)		148/58.5	2.5 (2.1 to 3.0)	6.2 (4.5 to 7.8)
AML	82/6.2	13.2 (10.5 to 16.4)	58.2 (44.5 to 71.8)		44/0.0	33.8 (23.8 to 43.7)		12/1.1	10.9 (5.6 to 19.0)	8.4 (3.2 to 13.6)		26/5.1	5.1 (3.3 to 7.5)	16.0 (8.4 to 23.7)
Hodgkin’s lymphoma	331/47.3	7.0 (6.3 to 7.8)	66.6 (58.2 to 75.0)		160/0.0	37.6 (31.7 to 43.4)		80/11.5	6.9 (5.5 to 8.6)	16.1 (12.0 to 20.2)		91/35.8	2.5 (2.0 to 3.1)	13.0 (8.6 to 17.3)
Non-Hodgkin’s lymphoma	131/31.9	4.1 (3.4 to 4.9)	32.7 (25.3 to 40.1)		48/0.0	15.8 (11.3 to 20.3)		31/8.2	3.8 (2.6 to 5.4)	7.5 (3.9 to 11.1)		52/23.7	2.2 (1.6 to 2.9)	9.3 (4.7 to 14.0)
Neuroblastoma	144/16.8	8.5 (7.2 to 10.1)	44.6 (36.4 to 52.9)		85/0.0	29.8 (23.5 to 36.2)		22/4.2	5.3 (3.3 to 8.0)	6.3 (3.0 to 9.5)		37/12.7	2.9 (2.1 to 4.0)	8.5 (4.3 to 12.7)
Non-heritable retinoblastoma	31/23.2	1.3 (0.9 to 1.9)	3.0 (−1.2 to 7.2)		0/0.0	0.0 (0.0 to 0.0)		15/6.8	2.2 (1.2 to 3.6)	3.1 (0.2 to 6.0)		16/16.4	1.0 (0.6 to 1.6)	−0.1 (−3.1 to 2.9)
Heritable retinoblastoma	138/16.9	8.2 (6.9 to 9.6)	60.1 (48.6 to 71.5)		21/0.0	10.4 (6.0 to 14.9)		99/4.7	21.0 (17.0 to 25.5)	46.8 (37.1 to 56.4)		18/12.2	1.5 (0.9 to 2.3)	2.9 (−1.2 to 7.0)
Wilms’s tumour	184/33.8	5.4 (4.7 to 6.3)	29.1 (24.0 to 34.3)		52/0.0	10.1 (7.3 to 12.8)		49/8.1	6.0 (4.5 to 8.0)	7.9 (5.3 to 10.6)		83/25.7	3.2 (2.6 to 4.0)	11.1 (7.6 to 14.6)
Bone sarcoma	198/24.6	8.0 (7.0 to 9.2)	79.5 (66.9 to 92.2)		135/0.0	61.9 (51.5 to 72.4)		37/7.4	5.0 (3.5 to 6.9)	13.6 (8.1 to 19.0)		26/17.2	1.5 (1.0 to 2.2)	4.0 (−0.5 to 8.6)
Soft tissue sarcoma	253/39.2	6.5 (5.7 to 7.3)	50.8 (43.4 to 58.2)		149/0.0	35.4 (29.7 to 41.1)		50/10.9	4.6 (3.4 to 6.0)	9.3 (6.0 to 12.6)		54/28.3	1.9 (1.4 to 2.5)	6.1 (2.7 to 9.5)
Other	205/49.5	4.1 (3.6 to 4.7)	27.8 (22.8 to 32.8)		79/0.0	14.1 (11.0 to 17.3)		54/15.2	3.6 (2.7 to 4.6)	6.9 (4.4 to 9.5)		72/34.4	2.1 (1.6 to 2.6)	6.7 (3.8 to 9.7)
P for heterogeneity*		<0.01	<0.01			<0.01			<0.01	<0.01			<0.01	<0.01
Age at diagnosis (years):														
0-4	1662/167.9	9.9 (9.4 to 10.4)	51.2 (48.5 to 54.0)		932/0.0	32.0 (29.9 to 34.0)		351/38.6	9.1 (8.2 to 10.1)	10.7 (9.5 to 12.0)		379/129.3	2.9 (2.6 to 3.2)	8.6 (7.3 to 9.9)
5-9	1351/128.6	10.5 (10.0 to 11.1)	74.9 (70.5 to 79.3)		865/0.0	53.0 (49.5 to 56.5)		187/31.1	6.0 (5.2 to 6.9)	9.6 (7.9 to 11.2)		299/97.5	3.1 (2.7 to 3.4)	12.3 (10.3 to 14.4)
10-14	1462/194.4	7.5 (7.1 to 7.9)	76.4 (71.8 to 80.9)		829/0.0	49.9 (46.5 to 53.3)		257/57.2	4.5 (4.0 to 5.1)	12.0 (10.1 to 13.9)		376/137.2	2.7 (2.5 to 3.0)	14.4 (12.1 to 16.7)
P for trend*		<0.01	<0.01			<0.01			0.03	0.63			0.03	0.43
Treatment period:														
<1970	1329/228.1	5.8 (5.5 to 6.1)	81.0 (75.8 to 86.3)		638/0.0	47.0 (43.3 to 50.6)		295/79.7	3.7 (3.3 to 4.2)	15.8 (13.4 to 18.3)		396/148.5	2.7 (2.4 to 2.9)	18.2 (15.3 to 21.1)
1970-79	1247/125.0	10.0 (9.4 to 10.5)	73.7 (69.1 to 78.2)		708/0.0	46.5 (43.1 to 49.9)		240/26.9	8.9 (7.8 to 10.1)	14.0 (12.0 to 16.0)		299/98.1	3.0 (2.7 to 3.4)	13.2 (11.0 to 15.4)
1980-89	942/85.0	11.1 (10.4 to 11.8)	55.2 (51.3 to 59.0)		591/0.0	38.0 (35.0 to 41.1)		138/13.1	10.6 (8.9 to 12.5)	8.0 (6.6 to 9.5)		213/71.9	3.0 (2.6 to 3.4)	9.1 (7.2 to 10.9)
1990-2006	957/52.9	18.1 (17.0 to 19.3)	51.0 (47.6 to 54.4)		689/0.0	38.9 (36.0 to 41.8)		122/7.3	16.6 (13.8 to 19.9)	6.5 (5.2 to 7.7)		146/45.5	3.2 (2.7 to 3.8)	5.7 (4.3 to 7.0)
P for trend*		<0.01	<0.01			<0.01			<0.01	0.11			0.70	<0.01
Follow-up (years):														
5-19	3115/156.8	19.9 (19.2 to 20.6)	75.7 (72.9 to 78.5)		2368/0.0	60.6 (58.2 to 63.0)		322/21.2	15.2 (13.6 to 16.9)	7.7 (6.8 to 8.6)		425/135.5	3.1 (2.8 to 3.4)	7.4 (6.4 to 8.4)
20-29	547/101.7	5.4 (4.9 to 5.8)	33.7 (30.2 to 37.2)		164/0.0	12.4 (10.5 to 14.3)		154/19.8	7.8 (6.6 to 9.1)	10.2 (8.3 to 12.0)		229/82.0	2.8 (2.4 to 3.2)	11.1 (8.9 to 13.4)
30-39	417/98.9	4.2 (3.8 to 4.6)	47.9 (41.9 to 54.0)		63/0.0	9.5 (7.1 to 11.8)		155/29.8	5.2 (4.4 to 6.1)	18.9 (15.2 to 22.5)		199/69.1	2.9 (2.5 to 3.3)	19.6 (15.4 to 23.7)
40-49	277/83.5	3.3 (2.9 to 3.7)	77.5 (64.4 to 90.5)		25/0.0	10.0 (6.1 to 13.9)		109/33.5	3.3 (2.7 to 3.9)	30.2 (22.0 to 38.4)		143/50.0	2.9 (2.4 to 3.4)	37.2 (27.9 to 46.6)
50-59	103/43.0	2.4 (2.0 to 2.9)	98.3 (65.7 to 130.9)		6/0.0	9.8 (2.0 to 17.7)		48/19.5	2.5 (1.8 to 3.3)	46.6 (24.4 to 68.9)		49/23.5	2.1 (1.5 to 2.8)	41.8 (19.3 to 64.3)
≥60	16/7.0	2.3 (1.3 to 3.7)	177.4 (23.0 to 331.9)		0/0.0	0.0 (0.0 to 0.0)		7/3.0	2.3 (0.9 to 4.8)	78.3 (−23.9 to 180.4)		9/4.0	2.3 (1.0 to 4.3)	99.1 (−16.7 to 215.0)
P for trend†		<0.01	<0.01			<0.01			<0.01	<0.01			0.24	<0.01
Attained age (years):														
5-19	2247/70.5	31.9 (30.6 to 33.2)	89.6 (85.7 to 93.4)		1835/0.0	75.5 (72.1 to 79.0)		184/10.1	18.2 (15.7 to 21.0)	7.2 (6.1 to 8.2)		228/60.4	3.8 (3.3 to 4.3)	6.9 (5.7 to 8.1)
20-29	1003/111.2	9.0 (8.5 to 9.6)	45.6 (42.4 to 48.8)		553/0.0	28.3 (25.9 to 30.6)		184/13.7	13.5 (11.6 to 15.6)	8.7 (7.3 to 10.1)		266/97.5	2.7 (2.4 to 3.1)	8.6 (7.0 to 10.2)
30-39	535/94.8	5.6 (5.2 to 6.1)	40.5 (36.4 to 44.7)		161/0.0	14.8 (12.5 to 17.1)		163/20.2	8.1 (6.9 to 9.4)	13.1 (10.8 to 15.5)		211/74.6	2.8 (2.5 to 3.2)	12.6 (9.9 to 15.2)
40-49	364/95.1	3.8 (3.4 to 4.2)	51.9 (44.6 to 59.1)		52/0.0	10.0 (7.3 to 12.8)		131/30.5	4.3 (3.6 to 5.1)	19.4 (15.1 to 23.7)		181/64.6	2.8 (2.4 to 3.2)	22.4 (17.4 to 27.5)
50-59	238/76.1	3.1 (2.7 to 3.6)	92.2 (75.0 to 109.5)		18/0.0	10.3 (5.5 to 15.0)		99/32.5	3.0 (2.5 to 3.7)	37.9 (26.8 to 49.0)		121/43.6	2.8 (2.3 to 3.3)	44.1 (31.8 to 56.4)
≥60	88/43.2	2.0 (1.6 to 2.5)	108.1 (63.7 to 152.5)		7/0.0	16.9 (4.4 to 29.4)		34/19.9	1.7 (1.2 to 2.4)	33.9 (6.4 to 61.5)		47/23.3	2.0 (1.5 to 2.7)	57.3 (24.8 to 89.7)
P for trend*		<0.01	<0.01			<0.01			<0.01	<0.01			<0.01	<0.01

SMR=standardised mortality ratio; AER=absolute excess risk; CNS=central nervous system; PNET=primitive neuroectodermal tumour, AML=acute myeloid leukaemia.

*P for heterogeneity or P for trend determined from multivariable Poisson model adjusting for sex, first primary neoplasm type, age at diagnosis, treatment period, and attained age.

†P for heterogeneity or P for trend determined from multivariable Poisson model adjusting for sex, first primary neoplasm type, age at diagnosis, treatment period, and follow-up.

**Table 4 tbl4:** Excess mortality ratios from univariable and multivariable Poisson models assessing the risk of all, recurrence or progression, subsequent primary neoplasm, and non-neoplastic causes of death by treatment period

Treatment period	All causes				Recurrence or progression				Subsequent primary neoplasms				Non-neoplastic causes		
	Observed/expected	Univariable: EMR (95% CI)	Multivariable*: EMR (95% CI)		Observed/expected	Univariable: EMR (95% CI)	Multivariable*: EMR (95% CI)		Observed/expected	Univariable: EMR (95% CI)	Multivariable*: EMR (95% CI)		Observed/expected	Univariable: EMR (95% CI)	Multivariable*: EMR (95% CI)
<1970	1329/228.1	1 (ref)	1 (ref)		638/0.0	1 (ref)	1 (ref)		295/79.7	1 (ref)	1 (ref)		396/148.5	1 (ref)	1 (ref)
1970-79	1247/125.0	0.9 (0.8 to 1.0)	0.8 (0.7 to 0.8)		708/0.0	1.0 (0.9 to 1.1)	0.7 (0.6 to 0.7)		240/26.9	0.9 (0.7 to 1.1)	1.4 (1.1 to 1.7)		299/98.1	0.7 (0.6 to 0.9)	1.0 (0.8 to 1.3)
1980-89	942/85.0	0.7 (0.6 to 0.7)	0.5 (0.5 to 0.6)		591/0.0	0.8 (0.7 to 0.9)	0.4 (0.4 to 0.5)		138/13.1	0.5 (0.4 to 0.6)	0.9 (0.7 to 1.2)		213/71.9	0.5 (0.4 to 0.6)	0.9 (0.7 to 1.2)
1990-2006	957/52.9	0.6 (0.6 to 0.7)	0.3 (0.3 to 0.4)		689/0.0	0.8 (0.7 to 0.9)	0.3 (0.2 to 0.3)		122/7.3	0.4 (0.3 to 0.5)	0.9 (0.7 to 1.2)		146/45.5	0.3 (0.2 to 0.4)	0.6 (0.4 to 0.8)
P for trend†		<0.01	<0.01			<0.01	<0.01			<0.01	0.10			<0.01	<0.01

EMR=excess mortality ratios.

*Adjusted for sex, first primary neoplasm diagnosis, age at diagnosis, and attained age.

†Calculated using likelihood ratio tests to assess the effect of treatment period.

When treatment period was further assessed, a statistically significant decline in excess mortality among those with a more recent diagnosis was observed for several types of first primary neoplasms, after adjusting for sex, age at diagnosis, and attained age: CNS neoplasms (excluding primitive neuroectodermal tumour) (P for trend <0.01), CNS primitive neuroectodermal tumour (P for trend <0.01), leukaemia (excluding acute myeloid leukaemia) (P for trend <0.01), acute myeloid leukaemia (P for trend 0.03), Hodgkin’s lymphoma (P for trend <0.01), non-Hodgkin’s lymphoma (P for trend 0.03), heritable retinoblastoma (P for trend 0.04), and other types of first primary neoplasms (P for trend 0.01, table 5). The largest declines in excess all cause mortality were observed among survivors of leukaemia (excluding acute myeloid leukaemia) and Hodgkin’s lymphoma, as survivors with a diagnosis from 1990 to 2006 experienced 10% (both excess mortality ratios 0.1, 95% confidence interval 0.1 to 0.1) of the number of excess deaths observed among survivors with a diagnosis before 1970. Survivors of CNS neoplasms (excluding primitive neuroectodermal tumour), CNS primitive neuroectodermal tumour, non-Hodgkin’s lymphoma, heritable retinoblastoma, and other types of first primary neoplasms also showed substantial declines in excess mortality, with at least a 50% (all excess mortality ratios ≤0.5) decline in excess all cause mortality among survivors with a diagnosis from 1990 to 2006 compared with survivors with a diagnosis before 1970.

**Table 5 tbl5:** Excess mortality ratios for all, recurrence or progression, subsequent primary neoplasm, and non-neoplastic causes of death, by first primary neoplastic type and treatment period

First primary neoplasm by treatment period	All causes			Recurrence or progression			Subsequent primary neoplasms			Non-neoplastic	
	Observed/expected	EMR (95% CI)		Observed/expected	EMR (95% CI)		Observed/expected	EMR (95% CI)		Observed/expected	EMR (95% CI)
CNS (excluding PNET):											
<1970	506/62.6	1.0 (ref)		275/0.0	1.0 (ref)		53/22.6	1.0 (ref)		178/40.0	1.0 (ref)
1970-79	335/27.2	0.8 (0.7 to 0.9)		193/0.0	0.7 (0.6 to 0.8)		41/6.2	1.6 (0.9 to 3.0)		101/20.9	1.0 (0.7 to 1.4)
1980-89	252/15.0	0.7(0.6 to 0.8)		165/0.0	0.6(0.5 to 0.7)		27/2.4	1.6(0.9 to 3.2)		60/12.6	0.9(0.6 to 1.3)
1990-2006	241/10.8	0.4(0.3 to 0.5)		175/0.0	0.3(0.3 to 0.4)		27/1.5	1.5(0.8 to 3.1)		39/9.3	0.5(0.3 to 0.9)
P for trend*		<0.01			<0.01			0.24			0.02
CNS PNET:											
<1970	106/6.6	1.0 (ref)		61/0.0	1.0 (ref)		27/2.1	1.0 (ref)		18/4.6	1.0 (ref)
1970-79	76/3.5	0.7(0.5 to 1.0)		39/0.0	0.5(0.4 to 0.8)		18/0.7	1.0(0.5 to 2.0)		19/2.8	1.9(0.7 to 4.8)
1980-89	67/2.7	0.6(0.4 to 0.8)		50/0.0	0.6(0.4 to 0.8)		8/0.4	0.5(0.2 to 1.1)		9/2.3	1.2(0.4 to 4.0)
1990-2006	91/1.8	0.5(0.3 to 0.6)		65/0.0	0.4(0.2 to 0.5)		19/0.2	0.9(0.4 to 2.0)		7/1.6	0.8(0.2 to 2.8)
P for trend*		<0.01			<0.01			0.54			0.69
Leukaemia (excluding AML):											
<1970	98/4.1	1.0 (ref)		94/0.0	1.0 (ref)		1/1.3	1.0 (ref)		3/2.8	1.0 (ref)
1970-79	414/29.2	0.3(0.2 to 0.4)		309/0.0	0.2(0.2 to 0.3)		50/5.9	8.5(0.1 to 503.4)		55/23.3	NP
1980-89	289/23.1	0.1(0.1 to 0.2)		207/0.0	0.1(0.1 to 0.1)		32/3.4	5.1(0.1 to 309.5)		50/19.6	NP
1990-2006	303/14.8	0.1(0.1 to 0.1)		220/0.0	0.1(0.0 to 0.1)		43/2.1	6.7(0.1 to 401.6)		40/12.7	NP
P for trend*		<0.01			<0.01			0.71			0.10
AML:											
<1970	2/0.6	1.0 (ref)		1/0.0	1.0 (ref)		1/0.2	1.0 (ref)		0/0.4	1.0 (ref)
1970-79	14/1.6	1.8(0.3 to 10.5)		7/0.0	0.8(0.1 to 6.9)		4/0.3	2.1(0.1 to 40.5)		3/1.3	NP
1980-89	36/2.0	2.3(0.4 to 13.5)		15/0.0	0.7(0.1 to 5.2)		7/0.3	2.7(0.1 to 58.4)		14/1.7	NP
1990-2006	30/2.0	0.9(0.1 to 5.3)		21/0.0	0.4(0.0 to 2.7)		0/0.3	NP		9/1.7	NP
P for trend*		0.03			0.02			0.13			0.24
Hodgkin’s lymphoma:											
<1970	156/19.2	1.0 (ref)		87/0.0	1.0 (ref)		33/6.5	1.0 (ref)		36/12.8	1.0 (ref)
1970-79	105/14.8	0.4(0.3 to 0.6)		40/0.0	0.2(0.2 to 0.4)		32/3.2	1.4(0.7 to 2.7)		33/11.6	0.8(0.3 to 1.8)
1980-89	42/9.1	0.2(0.1 to 0.2)		19/0.0	0.1(0.1 to 0.2)		10/1.4	0.6(0.3 to 1.7)		13/7.7	0.4(0.1 to 1.2)
1990-2006	28/4.2	0.1(0.1 to 0.1)		14/0.0	0.1(0.0 to 0.1)		5/0.5	0.6(0.2 to 2.0)		9/3.7	0.3(0.1 to 1.1)
P for trend*		<0.01			<0.01			0.27			0.03
Non-Hodgkin’s lymphoma:											
<1970	46/15.7	1.0 (ref)		12/0.0	1.0 (ref)		12/5.6	1.0 (ref)		22/10.2	1.0 (ref)
1970-79	27/6.5	0.9(0.5 to 1.7)		11/0.0	0.9(0.4 to 2.0)		9/1.3	4.1(0.7 to 23.9)		7/5.2	0.5(0.1 to 2.2)
1980-89	31/6.3	0.6(0.3 to 1.2)		11/0.0	0.5(0.2 to 1.1)		5/0.9	1.8(0.2 to 13.7)		15/5.5	0.7(0.2 to 3.0)
1990-2006	27/3.3	0.5(0.3 to 1.0)		14/0.0	0.4(0.2 to 0.9)		5/0.4	1.6(0.2 to 11.9)		8/2.9	0.3(0.0 to 2.7)
P for trend*		0.03			0.02			0.92			0.33
Neuroblastoma:											
<1970	49/10.1	1.0 (ref)		18/0.0	1.0 (ref)		11/3.1	1.0 (ref)		20/7.1	1.0 (ref)
1970-79	23/2.7	1.1(0.6 to 1.9)		13/0.0	1.1(0.6 to 2.4)		5/0.5	1.3(0.4 to 4.9)		5/2.3	0.8(0.2 to 3.0)
1980-89	29/2.5	0.8(0.5 to 1.5)		17/0.0	0.8(0.4 to 1.6)		4/0.4	1.0(0.2 to 4.4)		8/2.1	0.7(0.2 to 2.8)
1990-2006	43/1.5	0.8(0.5 to 1.4)		37/0.0	1.0(0.5 to 1.7)		2/0.3	0.6(0.1 to 4.1)		4/1.2	0.3(0.1 to 2.1)
P for trend*		0.33			0.76			0.70			0.25
Non-heritable retinoblastoma:											
<1970	19/17.0	1.0 (ref)		0/0.0	1.0 (ref)		13/5.8	1.0 (ref)		6/11.2	1.0 (ref)
1970-79	9/4.1	9.0(0.1 to 1357.2)		0/0.0	1.0		1/0.7	0.4(0.0 to 8.2)		8/3.4	NP
1980-89	2/1.4	13.2(0.0 to 6321.2)		0/0.0	1.0		1/0.2	2.0(0.1 to 50.7)		1/1.2	NP
1990-2006	1/0.8	NP		0/0.0	1.0		0/0.1	NP		1/0.6	NP
P for trend*		0.92			NA			0.62			0.76
Heritable retinoblastoma:											
<1970	96/13.2	1.0 (ref)		11/0.0	1.0 (ref)		71/4.1	1.0 (ref)		14/9.1	1.0 (ref)
1970-79	21/2.3	0.9(0.5 to 1.5)		3/0.0	0.7(0.2 to 2.5)		15/0.4	0.9(0.5 to 1.7)		3/1.9	NP
1980-89	17/1.0	1.0(0.6 to 1.9)		5/0.0	1.2(0.4 to 3.7)		11/0.1	1.0(0.5 to 2.0)		1/0.9	2.5(0.1 to 61.7)
1990-2006	4/0.4	0.3(0.1 to 0.8)		2/0.0	0.4(0.1 to 1.9)		2/0.1	0.2(0.0 to 1.0)		0/0.3	NP
P for trend*		0.04			0.42			0.09			1.00
Wilms’s tumour:											
<1970	75/16.6	1.0 (ref)		11/0.0	1.0 (ref)		29/5.2	1.0 (ref)		35/11.4	1.0 (ref)
1970-79	65/10.0	1.2(0.8 to 1.9)		19/0.0	1.2(0.6 to 2.5)		17/1.8	0.9(0.4 to 2.0)		29/8.2	1.6(0.7 to 3.5)
1980-89	29/4.9	0.8(0.4 to 1.4)		10/0.0	0.6(0.3 to 1.5)		3/0.7	0.2(0.0 to 1.3)		16/4.2	2.0(0.7 to 5.5)
1990-2006	15/2.3	0.5(0.2 to 1.0)		12/0.0	0.6(0.3 to 1.4)		0/0.4	NP		3/1.9	NP
P for trend*		0.05			0.08			0.02			0.69
Bone sarcoma:											
<1970	48/13.2	1.0 (ref)		25/0.0	1.0 (ref)		13/5.2	1.0 (ref)		10/8.0	1.0 (ref)
1970-79	41/5.3	0.9(0.5 to 1.4)		23/0.0	0.8(0.4 to 1.4)		10/1.3	1.3(0.4 to 4.3)		8/4.0	0.2(0.0 to 1349.8)
1980-89	44/3.9	0.8(0.5 to 1.3)		29/0.0	0.7(0.4 to 1.2)		11/0.7	1.6(0.5 to 5.1)		4/3.2	NP
1990-2006	65/2.3	0.8(0.5 to 1.2)		58/0.0	0.8(0.5 to 1.3)		3/0.3	0.4(0.1 to 1.9)		4/2.0	1.1(0.1 to 9.6)
P for trend*		0.35			0.59			0.39			0.75
Soft tissue sarcoma:											
<1970	60/22.9	1.0 (ref)		26/0.0	1.0 (ref)		13/8.0	1.0 (ref)		21/14.8	1.0 (ref)
1970-79	66/7.3	1.7(1.1 to 2.7)		34/0.0	1.4(0.8 to 2.3)		17/1.5	7.3(1.7 to 31.6)		15/5.8	3.0(0.4 to 24.0)
1980-89	53/5.6	1.1(0.7 to 1.7)		35/0.0	0.9(0.6 to 1.6)		9/0.9	4.7(0.9 to 23.7)		9/4.7	0.8(0.1 to 10.5)
1990-2006	74/3.4	1.1(0.7 to 1.7)		54/0.0	0.9(0.5 to 1.4)		11/0.5	6.8(1.3 to 35.1)		9/3.0	2.2(0.3 to 19.6)
P for trend*		0.50			0.20			0.04			0.66
Other:											
<1970	68/26.3	1.0 (ref)		17/0.0	1.0 (ref)		18/10.1	1.0 (ref)		33/16.2	1.0 (ref)
1970-79	51/10.5	0.9(0.6 to 1.5)		17/0.0	0.9(0.4 to 1.7)		21/2.9	1.8(0.7 to 4.4)		13/7.5	0.4(0.1 to 1.7)
1980-89	51/7.5	0.8(0.5 to 1.3)		28/0.0	1.1(0.6 to 2.1)		10/1.4	0.8(0.3 to 2.2)		13/6.1	0.4(0.1 to 1.7)
1990-2006	35/5.3	0.3(0.2 to 0.6)		17/0.0	0.3(0.2 to 0.7)		5/0.7	0.4(0.1 to 1.3)		13/4.5	0.6(0.2 to 2.2)
P for trend*		<0.01			<0.01			0.06			0.42

EMR=excess mortality ratio; CNS=central nervous system; PNET=primitive neuroectodermal tumour; AML=acute myeloid leukaemia; NA=not applicable; NP=not possible to reliably calculate owing to small numbers.

*P for trend determined from multivariable Poisson model adjusted for sex, age at diagnosis, treatment period, and attained age.

### Cause specific mortality

The standardised mortality ratio was significantly increased for all causes of death, except for deaths due to a mental disorder (table 2). Recurrence or progression of the original tumour was the leading cause of death, accounting for 65.9% of the excess deaths among survivors. Subsequent primary neoplasms and circulatory causes of death were the next largest contributors to excess deaths, accounting for 16.8% and 5.6% of the excess, respectively. As attained age increased, subsequent primary neoplasms and non-neoplastic causes increasingly accounted for excess mortality, at 41.1% and 47.8% of excess deaths among survivors aged 50-59, respectively, and 31.4% and 53.0% of excess deaths among survivors aged 60 or more, respectively (table 6). In particular, the main contributor to excess mortality with increased attained age was circulatory causes, which accounted for 36.8% of the total excess mortality and 69.4% of the excess non-neoplastic mortality among those aged 60 years or more.

**Table 6 tbl6:** Absolute excess risks for all, recurrences or progression, subsequent primary neoplasms, non-neoplastic, circulatory, respiratory, external, and other non-neoplastic causes of death by attained age and expressed as a percentage of the total excess risk

Causes of death	Attained age (years)																	
	5-19			20-29			30-39			40-49			50-59			≥60		
	Observed/expected	AER	% total	Observed/expected	AER	% total	Observed/expected	AER	% total	Observed/expected	AER	% total	Observed/expected	AER	% total	Observed/expected	AER	% total
Recurrence or progression	1835/0.0	75.5	84.3	553/0.0	28.3	62.1	161/0.0	14.8	36.5	52/0.0	10.0	19.3	18/0.0	10.3	11.2	7/0.0	16.9	15.6
Subsequent primary neoplasms	184/10.1	7.2	8.0	184/13.7	8.7	19.1	163/20.2	13.1	32.3	131/30.5	19.4	37.4	99/32.5	37.9	41.1	34/19.9	33.9	31.4
Non-neoplastic	228/60.4	6.9	7.7	266/97.5	8.6	18.9	211/74.6	12.6	31.1	181/64.6	22.4	43.2	121/43.6	44.1	47.8	47/23.3	57.3	53.0
Circulatory	30/3.5	1.1	1.2	56/7.6	2.5	5.5	63/13.6	4.6	11.4	68/21.8	8.9	17.1	55/20.0	19.9	21.6	28/11.5	39.8	36.8
Respiratory	44/3.4	1.7	1.9	35/3.7	1.6	3.5	34/3.7	2.8	6.9	27/4.3	4.4	8.4	20/4.8	8.7	9.4	4/4.3	−0.8	−0.7
External*	42/35.5	0.3	0.3	69/61.4	0.4	0.9	42/32.8	0.8	2.0	23/15.9	1.4	2.7	9/5.0	2.3	2.5	3/1.1	4.6	4.3
Other non-neoplastic†	112/18.0	3.9	4.4	106/24.7	4.2	9.2	72/24.5	4.4	10.8	63/22.6	7.8	14.9	37/13.8	13.2	14.3	12/6.3	13.7	12.7
All causes	2247/70.5	89.6	100.0	1003/111.2	45.6	100.0	535/94.8	40.5	100.0	364/95.1	51.9	100.0	238/76.1	92.2	100.0	88/43.2	108.1	100.0

AER=absolute excess risk.

*Includes deaths due to transportation accidents, falls, drowning, fire, suicide, etc.

†Includes nervous, infection, digestive, perinatal, endocrine, genitourinary, musculoskeletal, mental, blood, and other cause of death.

Further investigations into cause specific mortality were conducted for each cause of death, with at least 150 observed events. These were recurrence or progression, subsequent primary neoplasms, non-neoplastic causes overall, circulatory causes, respiratory causes, and external causes.

#### Deaths due to recurrence or progression

Among 3421 deaths due to cancer, 2626 (76.8%) were attributed to recurrence or progression of the original cancer, which equated to 42.3 (95% confidence interval 40.7 to 43.9) excess deaths per 10 000 person years (table 2). All types of first primary neoplasms, except non-heritable retinoblastoma, were found to have excess deaths due to recurrence or progression, but noticeable excesses were observed for survivors of CNS neoplasms (excluding primitive neuroectodermal tumour) (absolute excess risk 64.8), CNS primitive neuroectodermal tumour (115.0), leukaemia (excluding acute myeloid leukaemia) (57.1), and bone sarcoma (61.9), where more than 50 excess deaths per 10 000 person years were observed (table 3). With regards to treatment period, the absolute excess risk significantly decreased among those treated more recently (P for trend <0.01) (table 4); compared with survivors with a diagnosis before 1970, those with a diagnosis from 1990 to 2006 experienced 30% (excess mortality ratio 0.3, 95% confidence interval 0.2 to 0.3) of the excess deaths due to recurrence or progression, after adjusting for sex, type of first primary neoplasm, age at diagnosis, and attained age.

When treatment period was assessed by type of first primary neoplasm, survivors of CNS neoplasms (excluding primitive neuroectodermal tumour) (P for trend <0.01), CNS primitive neuroectodermal tumour (P for trend <0.01), leukaemia (excluding acute myeloid leukaemia) (P for trend <0.01), acute myeloid leukaemia (P for trend 0.02), Hodgkin’s lymphoma (P for trend <0.01), non-Hodgkin’s lymphoma (P for trend 0.02), and other types of first primary neoplasm (P for trend <0.01) were found to have significantly fewer excess numbers of deaths among those with a most recent diagnosis, after adjustment (table 5). The strongest decline in the excess number of deaths due to recurrence or progression was observed for survivors of leukaemia (excluding acute myeloid leukaemia) and Hodgkin’s lymphoma, as survivors with a diagnosis from 1990 to 2006 experienced 10% (both excess mortality ratios 0.1, 95% confidence interval 0.0 to 0.1) of the excess number of deaths due to recurrence or progression observed among those with a diagnosis before 1970; for both types of first primary neoplasm the strongest decline in excess number of deaths due to recurrence or progression was observed from the treatment period before 1970 to the treatment period of 1970-79. Survivors of CNS neoplasms (excluding primitive neuroectodermal tumour) with a diagnosis from 1990 to 2006 experienced 30% (excess mortality ratio 0.3, 95% confidence interval 0.3 to 0.4) of the excess number of deaths due to recurrence or progression observed among those with a diagnosis before 1970; the corresponding percentage for survivors of CNS primitive neuroectodermal tumour (0.4, 0.2 to 0.5), acute myeloid leukaemia (0.4, 0.0 to 2.7), and non-Hodgkin’s lymphoma (0.4, 0.2 to 0.9) was 40%.

#### Deaths due to subsequent primary neoplasms

Survivors of childhood cancer were 6.3 times (95% confidence interval 5.8 to 6.7) more at risk of death due to a subsequent primary neoplasm than expected in the general population (table 2). Survivors of CNS primitive neuroectodermal tumour and heritable retinoblastoma had the greatest risk of death related to subsequent primary neoplasms, with standardised mortality ratios of 21.6 (95% confidence interval 16.9 to 27.2) and 21.0 (17.0 to 25.5), respectively (table 3). After adjusting for sex, type of first primary neoplasm, age at diagnosis, and attained age, there was no statistical evidence of an overall decline in the excess numbers of deaths from subsequent primary neoplasms with more recent treatment period (P for trend 0.10, table 4).

After adjustment, survivors of Wilms’s tumour (P for trend 0.02) with a more recent diagnosis experienced a lower number of excess deaths due to subsequent primary neoplasms (table 5). Conversely, among survivors of soft tissue sarcoma, the excess number of deaths from subsequent primary neoplasms increased among those with a more recent diagnosis (P for trend 0.04); more specifically, survivors of soft tissue sarcoma diagnosed during 1970-79, 1980-89, and 1990-2006 experienced 7.3 times (95% confidence interval 1.7 to 31.6), 4.7 times, (0.9 to 23.7), and 6.8 times (1.3 to 35.1) more excess deaths due to subsequent primary neoplasms than those with a diagnosis before 1970, respectively. A significant positive or negative trend for excess number of deaths related to subsequent primary neoplasms was not observed with treatment period for any other type of first primary neoplasm (all P for trend >0.05).

#### Deaths due to non-neoplastic causes

Survivors of childhood cancer were 2.9 times (95% confidence interval 2.7 to 3.1) more likely to die from a non-neoplastic cause of death than expected from the general population, which equated to 11.1 (95% confidence interval 10.1 to 12.1) excess deaths due to non-neoplastic causes per 10 000 person years (table 2). Survivors of acute myeloid leukaemia, CNS primitive neuroectodermal tumour, and CNS neoplasms (excluding primitive neuroectodermal tumour) were at greatest risk of death due to non-neoplastic causes, with standardised mortality ratios of 5.1 (95% confidence interval 3.3 to 7.5), 4.7 (3.5 to 6.2), and 4.6 (4.1 to 5.0), respectively (table 3). The number of excess deaths due to non-neoplastic causes among survivors was also observed to decrease in more recent treatment periods (P for trend <0.01), after adjusting for sex, type of first primary neoplasm, age at diagnosis, and attained age (table 4); survivors with a diagnosis from 1990 to 2006 experienced 60% (excess mortality ratio 0.6, 95%confidence interval 0.4 to 0.8) of the excess number of deaths due to non-neoplastic causes observed among survivors with a diagnosis before 1970.

When treatment period was further assessed by type of first primary neoplasm, only survivors of CNS neoplasms (excluding primitive neuroectodermal tumour) (P for trend 0.02) and Hodgkin’s lymphoma (P for trend=0.03) were found to have a significant decrease in excess mortality due to non-neoplastic causes among those treated more recently, after adjustment (table 5). Survivors of CNS neoplasms (excluding primitive neuroectodermal tumour) with a diagnosis from 1990 to 2006 experienced 50% (excess mortality ratio 0.5, 95% confidence interval 0.3 to 0.9) of the excess number of deaths due to non-neoplastic causes observed among survivors with a diagnosis before 1970, whereas the corresponding percentage for survivors of Hodgkin’s lymphoma was 30% (0.3, 0.1 to 1.1).

#### Deaths due to circulatory causes

Circulatory causes accounted for the largest number of deaths due to non-neoplastic causes, with 300 observed events (table 2). Survivors were 3.8 times (95% confidence interval 3.4 to 4.3) more at risk of death due to circulatory causes than expected from the general population, which equated to 3.6 (95% confidence interval 3.0 to 4.1) excess deaths due to circulatory causes per 10 000 person years. The risk of death due to circulatory causes was substantially increased (standardised mortality ratio ≥5) for survivors of acute myeloid leukaemia (16.6), CNS primitive neuroectodermal tumour (6.8), Wilms’s tumour (5.8), and Hodgkin’s lymphoma (5.0) (table 7). In the multivariable Poisson model there was no statistical evidence of a decline in excess numbers of death from circulatory causes with more recent treatment period (P for trend 0.19), after adjusting for sex, type of first primary neoplasm, age at diagnosis, and attained age (table 8).

**Table 7 tbl7:** Standardised mortality ratios and absolute excess risks for deaths due to circulatory, respiratory, and external causes, by potential explanatory factors

Factors	Circulatory causes				Respiratory causes				External causes*		
	Observed/expected	SMR (95% CI)	AER (95% CI)		Observed/expected	SMR (95% CI)	AER (95% CI)		Observed/expected	SMR (95% CI)	AER (95% CI)
Overall	300/78.0	3.8 (3.4 to 4.3)	3.6 (3.0 to 4.1)		164/24.2	6.8 (5.8 to 7.9)	2.3 (1.8 to 2.7)		188/151.7	1.2 (1.1 to 1.4)	0.6 (0.2 to 1.0)
Male	177/56.8	3.1 (2.7 to 3.6)	3.6 (2.8 to 4.3)		98/14.9	6.6 (5.3 to 8.0)	2.5 (1.9 to 3.0)		133/122.9	1.1 (0.9 to 1.3)	0.3 (−0.4 to 1.0)
Female	123/21.2	5.8 (4.8 to 6.9)	3.6 (2.8 to 4.4)		66/9.3	7.1 (5.5 to 9.0)	2.0 (1.4 to 2.6)		55/28.8	1.9 (1.4 to 2.5)	0.9 (0.4 to 1.4)
P for heterogeneity†		0.01	0.64			0.61	0.17			0.01	0.31
First primary neoplasm type:											
CNS (excluding PNET)	89/20.1	4.4 (3.5 to 5.4)	5.5 (4.0 to 7.0)		72/6.1	11.9 (9.3 to 15.0)	5.3 (4.0 to 6.6)		64/31.9	2.0 (1.5 to 2.6)	2.6 (1.3 to 3.8)
CNS PNET	15/2.2	6.8 (3.8 to 11.2)	6.8 (2.8 to 10.9)		15/0.7	22.6 (12.7 to 37.4)	7.7 (3.6 to 11.7)		7/5.1	1.4 (0.6 to 2.8)	1.0 (−1.7 to 3.8)
Leukaemia (excluding AML)	22/7.1	3.1 (1.9 to 4.7)	1.0 (0.4 to 1.7)		23/2.7	8.5 (5.4 to 12.7)	1.4 (0.7 to 2.0)		29/30.4	1.0 (0.6 to 1.4)	−0.1 (−0.8 to 0.6)
AML	11/0.7	16.6 (8.3 to 29.7)	7.9 (2.9 to 12.9)		3/0.2	12.6 (2.6 to 36.7)	2.1 (−0.5 to 4.7)		4/2.6	1.5 (0.4 to 3.9)	1.1 (−2.0 to 4.1)
Hodgkin’s lymphoma	42/8.5	5.0 (3.6 to 6.7)	7.9 (4.9 to 10.9)		7/2.3	3.1 (1.2 to 6.4)	1.1 (−0.1 to 2.3)		15/14.8	1.0 (0.6 to 1.7)	0.0 (−1.7 to 1.8)
Non-Hodgkin’s lymphoma	24/5.7	4.2 (2.7 to 6.2)	6.0 (2.9 to 9.2)		5/1.6	3.1 (1.0 to 7.4)	1.1 (−0.3 to 2.6)		11/9.6	1.1 (0.6 to 2.0)	0.4 (−1.7 to 2.6)
Neuroblastoma	8/2.5	3.3 (1.4 to 6.4)	1.9 (−0.0 to 3.9)		6/0.8	7.2 (2.6 to 15.7)	1.8 (0.1 to 3.5)		12/5.4	2.2 (1.1 to 3.9)	2.3 (−0.1 to 4.7)
Non-heritable retinoblastoma	2/4.1	0.5 (0.1 to 1.8)	−0.8 (−1.9 to 0.3)		1/1.3	0.8 (0.0 to 4.3)	−0.1 (−0.9 to 0.6)		4/5.9	0.7 (0.2 to 1.7)	−0.7 (−2.2 to 0.8)
Heritable retinoblastoma	5/2.8	1.8 (0.6 to 4.2)	1.1 (−1.1 to 3.3)		4/0.9	4.5 (1.2 to 11.4)	1.5 (−0.4 to 3.5)		2/4.8	0.4 (0.1 to 1.5)	−1.4 (−2.7 to 0.0)
Wilms’s tumour	27/4.7	5.8 (3.8 to 8.4)	4.3 (2.4 to 6.3)		7/1.6	4.4 (1.8 to 9.1)	1.1 (0.0 to 2.1)		16/11.4	1.4 (0.8 to 2.3)	0.9 (−0.6 to 2.4)
Bone Sarcoma	12/4.6	2.6 (1.4 to 4.6)	3.4 (0.3 to 6.5)		1/1.4	0.7 (0.0 to 4.1)	−0.2 (−1.1 to 0.7)		5/6.1	0.8 (0.3 to 1.9)	−0.5 (−2.5 to 1.5)
Soft tissue sarcoma	16/6.9	2.3 (1.3 to 3.8)	2.2 (0.3 to 4.0)		11/2.1	5.4 (2.7 to 9.6)	2.1 (0.6 to 3.7)		9/10.9	0.8 (0.4 to 1.6)	−0.5 (−1.9 to 0.9)
Other	27/8.2	3.3 (2.2 to 4.8)	3.4 (1.5 to 5.2)		9/2.7	3.3 (1.5 to 6.3)	1.1 (0.1 to 2.2)		10/12.7	0.8 (0.4 to 1.4)	−0.5 (−1.6 to 0.6)
P for heterogeneity†		0.01	0.01			0.01	0.01			0.01	0.01
Age at diagnosis (years):											
0-4	90/22.4	4.0 (3.2 to 4.9)	2.3 (1.7 to 3.0)		64/7.8	8.2 (6.3 to 10.4)	1.9 (1.4 to 2.5)		69/58.6	1.2 (0.9 to 1.5)	0.4 (−0.2 to 0.9)
5-9	83/19.2	4.3 (3.4 to 5.4)	3.9 (2.8 to 5.0)		43/6.0	7.2 (5.2 to 9.7)	2.3 (1.5 to 3.1)		46/43.1	1.1 (0.8 to 1.4)	0.2 (−0.6 to 1.0)
10-14	127/36.4	3.5 (2.9 to 4.2)	5.5 (4.1 to 6.8)		57/10.4	5.5 (4.1 to 7.1)	2.8 (1.9 to 3.7)		73/50.0	1.5 (1.1 to 1.8)	1.4 (0.4 to 2.4)
P for trend†		0.04	0.27			0.26	0.74			0.26	0.15
Treatment period:											
<1970	145/50.0	2.9 (2.4 to 3.4)	7.0 (5.3 to 8.7)		61/14.3	4.3 (3.3 to 5.5)	3.4 (2.3 to 4.6)		65/40.9	1.6 (1.2 to 2.0)	1.8 (0.6 to 2.9)
1970-79	74/16.7	4.4 (3.5 to 5.6)	3.8 (2.7 to 4.9)		48/5.2	9.3 (6.8 to 12.3)	2.8 (1.9 to 3.7)		55/45.2	1.2 (0.9 to 1.6)	0.6 (−0.3 to 1.6)
1980-89	63/7.8	8.1 (6.2 to 10.3)	3.6 (2.5 to 4.6)		36/2.9	12.4 (8.7 to 17.2)	2.1 (1.4 to 2.9)		41/38.9	1.1 (0.8 to 1.4)	0.1 (−0.7 to 0.9)
1990-2006	18/3.5	5.2 (3.1 to 8.2)	0.8 (0.4 to 1.3)		19/1.8	10.4 (6.2 to 16.2)	1.0 (0.5 to 1.5)		27/26.7	1.0 (0.7 to 1.5)	0.0 (−0.6 to 0.6)
P for trend†		0.25	0.18			0.3	0.01			0.07	0.01
Follow-up (years):											
5-19	70/9.7	7.2 (5.6 to 9.1)	1.5 (1.1 to 2.0)		69/6.4	10.8 (8.4 to 13.7)	1.6 (1.2 to 2.0)		99/82.4	1.2 (1.0 to 1.5)	0.4 (−0.1 to 0.9)
20-29	70/13.0	5.4 (4.2 to 6.8)	4.3 (3.1 to 5.6)		33/3.8	8.7 (6.0 to 12.2)	2.2 (1.4 to 3.1)		44/40.0	1.1 (0.8 to 1.5)	0.3 (−0.7 to 1.3)
30-39	67/20.9	3.2 (2.5 to 4.1)	6.9 (4.5 to 9.4)		32/4.4	7.2 (5.0 to 10.2)	4.2 (2.5 to 5.8)		29/20.2	1.4 (1.0 to 2.1)	1.3 (−0.3 to 2.9)
40-49	63/21.3	3.0 (2.3 to 3.8)	16.7 (10.5 to 22.9)		21/5.1	4.1 (2.5 to 6.3)	6.4 (2.8 to 10.0)		14/7.3	1.9 (1.0 to 3.2)	2.7 (−0.3 to 5.6)
50-59	27/11.2	2.4 (1.6 to 3.5)	25.9 (9.3 to 42.6)		8/3.7	2.1 (0.9 to 4.2)	7.0 (−2.1 to 16.1)		2/1.7	1.2 (0.1 to 4.3)	0.5 (−4.0 to 5.0)
≥60	3/1.9	1.6 (0.3 to 4.6)	21.5 (−45.4 to 88.4)		1/0.8	1.2 (0.0 to 6.9)	3.9 (−34.7 to 42.5)		0/0.1	0.0 (0.0 to 0.0)	−3.0 (−3.0 to 3.0)
P for trend‡		<0.01	<0.01			<0.01	<0.01			<0.01	<0.01
Attained age (years):											
5-19	30/3.5	8.7 (5.8 to 12.4)	1.1 (0.7 to 1.5)		44/3.4	13.0 (9.4 to 17.4)	1.7 (1.1 to 2.2)		42/35.5	1.2 (0.9 to 1.6)	0.3 (−0.3 to 0.8)
20-29	56/7.6	7.3 (5.5 to 9.5)	2.5 (1.7 to 3.2)		35/3.7	9.3 (6.5 to 13.0)	1.6 (1.0 to 2.2)		69/61.4	1.1 (0.9 to 1.4)	0.4 (−0.4 to 1.2)
30-39	63/13.6	4.6 (3.6 to 5.9)	4.6 (3.1 to 6.0)		34/3.7	9.2 (6.4 to 12.8)	2.8 (1.7 to 3.8)		42/32.8	1.3 (0.9 to 1.7)	0.8 (−0.3 to 2.0)
40-49	68/21.8	3.1 (2.4 to 3.9)	8.9 (5.8 to 12.0)		27/4.3	6.3 (4.2 to 9.2)	4.4 (2.4 to 6.3)		23/15.9	1.4 (0.9 to 2.2)	1.4 (−0.4 to 3.2)
50-59	55/20.0	2.7 (2.1 to 3.6)	19.9 (11.7 to 28.2)		20/4.8	4.2 (2.5 to 6.4)	8.7 (3.7 to 13.7)		9/5.0	1.8 (0.8 to 3.4)	2.3 (−1.1 to 5.6)
≥60	28/11.5	2.4 (1.6 to 3.5)	39.8 (14.8 to 64.8)		4/4.3	0.9 (0.3 to 2.4)	−0.8 (−10.3 to 8.6)		3/1.1	2.7 (0.6 to 8.0)	4.6 (−3.6 to 12.8)
P for trend†		0.01	0.01			0.01	0.04			0.79	0.08

SMR=standardised mortality ratio; AER=absolute excess risk; CNS=central nervous system; PNET=primitive neuroectodermal tumour, AML=acute myeloid leukaemia.

*Includes deaths due to transportation accidents, falls, drowning, fire, suicide, etc.

†P for heterogeneity or P for trend determined from multivariable Poisson model adjusting for sex, first primary neoplasm type, age at diagnosis, treatment period, and attained age.

‡P for heterogeneity or P for trend determined from multivariable Poisson model adjusting for sex, first primary neoplasm type, age at diagnosis, treatment period, and follow-up.

**Table 8 tbl8:** Excess mortality ratios from univariable and multivariable Poisson models assessing the risk of circulatory, respiratory, and external causes of death by treatment period

Treatment period	Circulatory causes				Respiratory causes				External causes*		
	Observed/expected	Univariable: EMR (95% CI)	Multivariable†: EMR (95% CI)		Observed/expected	Univariable: EMR (95% CI)	Multivariable†: EMR 95% CI)		Observed/expected	Univariable: EMR (95% CI)	Multivariable†: EMR (95% CI)
<1970	145/50.0	1 (ref)	1 (ref)		61/14.3	1 (ref)	1 (ref)		65/40.9	1 (ref)	1 (ref)
1970-79	74/16.7	0.5 (0.3 to 0.8)	0.9 (0.6 to 1.4)		48/5.2	0.8 (0.5 to 1.3)	1.0 (0.7 to 1.7)		55/45.2	0.4 (0.1 to 1.8)	0.7 (0.3 to 1.7)
1980-89	63/7.8	0.5 (0.3 to 0.7)	1.3 (0.8 to 2.0)		36/2.9	0.6 (0.4 to 1.0)	0.9 (0.5 to 1.6)		41/38.9	0.1 (0.0 to 33.7)	0.5 (0.2 to 1.5)
1990-2006	18/3.5	0.1 (0.1 to 0.2)	0.4 (0.2 to 0.6)		19/1.8	0.3 (0.2 to 0.5)	0.4 (0.2 to 0.8)		27/26.7	0.0 (0.0 to 0.0)	0.0 (0.0 to 0.0)
P for trend‡		<0.01	0.19			<0.01	0.01			<0.01	<0.01

EMR=excess mortality ratios.

*Includes deaths due to transportation accidents, falls, drowning, fire, suicide, etc.

†Adjusted for sex, first primary neoplasm type, age at diagnosis, and attained age.

‡Calculated using likelihood ratio tests to assess the effect of treatment period.

#### Death due to respiratory causes

Deaths due to respiratory causes occurred 6.8 times (95% confidence interval 5.8 to 7.9) more than that expected from the general population (table 2). A substantial excess risk (standardised mortality ratio ≥5) was observed among survivors of CNS primitive neuroectodermal tumour (22.6), acute myeloid leukaemia (12.6), CNS (excluding primitive neuroectodermal tumour) (11.9), leukaemia (excluding acute myeloid leukaemia) (8.5), neuroblastoma (7.2), and soft tissue sarcoma (5.4) (table 7). After adjusting for sex, type of first primary neoplasm, age at diagnosis, and attained age, a statistically significant decline (P for trend 0.01) in excess number of deaths was observed among those treated more recently (table 8); compared with survivors who received a diagnosis before 1970, survivors who received a diagnosis from 1990 to 2006 experienced 40% (excess mortality ratio 0.4, 95% confidence interval 0.2 to 0.8) of the excess number of deaths due to respiratory causes.

#### Deaths due to external causes

Survivors of childhood cancer had a slight increased risk of death due to external causes compared with that expected from the general population, with a standardised mortality ratio of 1.2 (95% confidence interval 1.1 to 1.4) (table 2). Only survivors of CNS neoplasms (excluding primitive neuroectodermal tumour) (2.0, 1.5 to 2.6) and neuroblastoma (2.2, 1.1 to 3.9) had a significant increased risk compared with that expected from the general population (table 7). With regards to treatment period, the number of excess deaths due to external causes significantly declined (P for trend <0.01) among those treated more recently, with those treated from 1990 to 2006 having no observed excess risk, after adjusting for sex, type of first primary neoplasm, age at diagnosis, and attained age (table 8).

## Discussion 

This study of late mortality after childhood cancer within a cohort of 34 489 five year survivors, among whom 4475 deaths were observed, provides an opportunity to investigate the impact of treatment period (1940-2006) on the risk of specific causes of death and the pattern of excess deaths among survivors aged at least 50 years. Previously we have reported the risk of cause specific death after childhood cancer within the same cohort.^2^ However, this updated analysis includes an additional 16 509 five year survivors, and adds a further 1434 deaths and 250 728 person years, thus exceeding considerably the numbers available in our previous study and comparable studies by the CCSS,^7^ SEER,^8^ and Nordic countries.^3^ Our methodological focus is different to these previous studies, as they concentrated on either cumulative risks, which ignore expected mortality when assessing differences in curves, or standardised mortality ratios, which being a measure of relative risk relate to a baseline risk that is often unclear. We concentrated on the absolute excess risk, which is an excess number of observed deaths beyond those expected from the general population, and so is directly interpretable in terms of adverse health impact on survivors.

Our findings indicate that the net effect of more modern cancer treatment, and increased surveillance and treatment of late effects, which were more commonly available among survivors treated more recently, was to reduce excess mortality. The number of excess deaths from all causes declined among those treated more recently, in that those treated from 1990 to 2006 had 30% of the excess number experienced by those treated before 1970. The corresponding percentages for the number of excess deaths from recurrence or progression and non-neoplastic causes were 30% and 60%, respectively.

Previous literature has suggested that late mortality was higher in earlier treatment periods (before 1970) than in more recent times (1970 to 2016).^14^ ^15^ ^16^ ^17^ Three previous large cohorts from SEER,^8^ the CCSS,^7^ and the Nordic countries^3^ have reported on treatment period effects among survivors of childhood cancer, where in all three cohorts observed there was a significant decline in cumulative mortality from all causes and deaths due to recurrence or progression for those with a more recent diagnosis. The Nordic countries also reported a statistically significant decline in deaths due to non-neoplastic causes and no treatment period effect for deaths due to subsequent primary neoplasms,^3^ whereas the CCSS also reported significant declines for subsequent primary neoplasms and for cardiac and respiratory causes.^7^ Our findings confirm those of these three studies, as well as smaller reports that investigated treatment period trends in Scotland^4^ and Canada,^9^ as we too found a decline in excess mortality for deaths due to all causes, recurrence or progression, and non-neoplastic causes. We did not observe a decline in the excess numbers of deaths from subsequent primary neoplasms or from circulatory causes overall, as reported by the CCSS,^7^ but when we restricted the period of diagnosis within the BCCSS cohort from 1970 to 1999 (to match the CCSS) there was evidence of a decline in the excess number of deaths from both subsequent primary neoplasms and circulatory causes among those with a more recent diagnosis; the excess number of deaths from subsequent primary neoplasm and circulatory causes among those with a diagnosis from 1995 to 1999 was 48% and 21% of those observed among those with a diagnosis from 1970 to 1975, respectively.

When we assessed trends in excess deaths with treatment period by first primary diagnosis, most types of first primary neoplasm (CNS (excluding primitive neuroectodermal tumour), CNS primitive neuroectodermal tumour, leukaemia (excluding acute myeloid leukaemia), acute myeloid leukaemia, Hodgkin’s lymphoma, non-Hodgkin’s lymphoma, heritable retinoblastoma, and other first primary neoplasms) experienced significantly fewer excess deaths from all causes after treatment from 1990 to 2006 compared with treatment before 1970. These findings add to the literature, which to date has only identified significant declines with treatment period in all cause mortality for survivors of leukaemia,^3^ ^7^ ^8^ Hodgkin’s lymphoma,^3^ ^7^ ^8^ non-Hodgkin’s lymphoma,^7^ ^8^ rhabdomyosarcoma,^8^ neuroblastoma,^7^ and some CNS cancers.^3^ ^7^ As observed in SEER,^8^ CCSS,^7^ and the Nordic studies,^3^ this reduction was largely due to decreasing mortality from recurrence or progression in those with a more recent diagnosis, and suggests that survivors of treatment in more recent periods have more durable remissions, or that the treatment for relapse, recurrence, or progression of the first primary neoplasm has improved more recently. When we assessed deaths due to subsequent primary neoplasm for treatment period effects, survivors of Wilms’s tumour and soft tissue sarcoma experienced significantly less and significantly more excess deaths due to subsequent primary neoplasm, respectively, among those with a more recent diagnosis. This is inconsistent with the Nordic report, as only survivors of CNS cancer were identified as having a statistically significant decline in excess deaths due to subsequent primary neoplasm with treatment period.^3^ We additionally identified statistically significant declines in deaths due to non-neoplastic causes for survivors of CNS neoplasms (excluding primitive neuroectodermal tumour) and Hodgkin’s lymphoma. Although the Nordic countries also found a statistically significant decline in non-neoplastic mortality for survivors of Hodgkin’s lymphoma, a significant decrease was also observed for survivors of leukaemia, which was not observed in the current study.^3^ Potential explanations for the differences observed between studies may relate to the number of deaths observed for each type of first primary neoplasm in each treatment period, age definition of childhood cancer, available follow-up time, length of diagnosis period assessed, and differences in treatment regimens utilised—the last of which has been clearly documented for survivors of acute myeloid leukaemia.^18^ ^19^ However, as our study included more deaths, longer follow-up, and a wider period of diagnosis, there is greater statistical power for detecting decreases in cause specific mortality.

This study also assessed late mortality among a population of survivors of childhood cancer in their sixth and seventh decades of life. Among those aged at least 50 years, the percentage of excess deaths due to non-neoplastic causes was about 50%, and this is likely to be an underestimate for two reasons. Firstly, bias in death certification inflating deaths from recurrence or progression is well known.^20^ Secondly, when we were uncertain about a particular cause of death we assigned it to recurrence or progression. Interestingly, among those aged 50-59 years, 41% and 22% of excess deaths were due to subsequent primary neoplasms and circulatory causes, respectively, whereas the corresponding percentages for those aged 60 years or more were 31% and 37%. This study provides evidence that as survivors age beyond 60 years, circulatory causes account for more excess deaths than subsequent primary neoplasms. This is not entirely surprising because in the general population, circulatory conditions account for substantially more deaths than neoplasms among those aged 60 years or more. Excess deaths due to non-neoplastic causes are likely attributable to late complications of treatment, as radiotherapy and chemotherapy have been associated with adverse circulatory,^21^ ^22^ ^23^ ^24^ ^25^ respiratory,^26^ ^27^ ^28^ endocrine,^29^ ^30^ neurological,^31^ ^32^ and other chronic health conditions.^29^ ^33^

### Putting the results into context

Over the treatment periods covered by this study, survival after almost all specific types of childhood cancer has improved substantially. For all childhood cancers combined, survival to five years has increased from 28% to 77% among those with a diagnosis before 1970 and with a diagnosis from 1996 to 2000, respectively.^34^ Successful treatment among those with a diagnosis before 1970 was attributable to surgery or radiotherapy, or both. Thereafter, chemotherapy of increasing complexity was introduced, leading to substantial improvements in survival of most types of childhood cancer not successfully treatable before 1970. In the 1990s bone marrow transplantation with high dose chemotherapy and often total body irradiation was introduced for those types of malignancy not previously responsive to conventional radiotherapy and chemotherapy, either as first line treatment or as second line treatment. Consequently, the proportion of those with a diagnosis of cancer in childhood surviving to five years has increased substantially, but the intensity of treatment has in general increased to achieve this success. This raises two important questions. Firstly, are those reaching five year survival as a result of the increasingly aggressive treatments in more recent decades truly cured, or is there evidence that recurrence or progression is merely postponed? Secondly, is the more aggressive treatment introduced in more recent decades associated with an increase in number of excess deaths from further primary cancers or non-neoplastic causes among five year survivors? The evidence presented in table 7 is overwhelmingly reassuring in relation to both questions. Firstly, the excess number of deaths attributable to recurrence or progression subsequent to five year survival substantially declines among those treated from 1990 to 2006, compared with those treated before 1970, for all CNS tumours, all types of leukaemia, and all types of lymphoma; for each of these diagnostic groups the excess number of deaths attributable to the original cancer is at most 40% among those treated from 1990 to 2006 compared with those treated before 1970. Secondly, with the exception of further cancers after soft tissue sarcoma, there is no evidence that the excess number of deaths attributable to either further primary cancers or non-neoplastic causes among five year survivors of each specific childhood cancer treated in more recent decades exceeds that observed among those treated earlier (table 7). In the past three decades, through recruitment to randomised clinical trials, there have been systematic efforts to reduce the risk of adverse health outcomes experienced by survivors of types of childhood cancer with good prognosis by modifying treatment regimens with the aim of maintaining the levels of cure, but reducing the risk of long term toxicity. It is likely that such efforts have contributed to the absence of an increase in excess numbers of deaths either from further primary cancers or from non-neoplastic causes, for the specific childhood cancers reported in table 7, in relation to treatment period.

### Strengths and weaknesses of this study

This study of mortality in five year survivors of childhood cancer provides the most precise estimates of risk to date owing to the fact it includes more survivors and observed deaths than previously reported. Because of our study’s population based design, selection bias is minimised and results are generalisable to Great Britain. By including survivors who received a diagnosis across almost seven decades, our study provides an opportunity to assess mortality from the pre-chemotherapy period to modern treatments and protocols. Furthermore, owing to our long follow-up time, we provide results on the risk of mortality beyond 60 years of age. By aggregating these strengths, the results presented in this study provide evidence that the net impact of more modern treatments and associated care is to reduce the excess numbers of subsequent deaths overall, from recurrence or progression and from non-neoplastic causes.

A weakness of our study is the absence of detailed information on treatment, which prevented investigation of treatment types. Another potential weakness of this study is that vital status was obtained through data linkage. Although we used NHS number, first name, middle initial, current surname, and date of birth to link our cohort with the national death registry, unsuccessful and incorrect linkage is possible. Unsuccessful attempts should be limited, however, as in our most recent linkage of approximately 16 500 survivors with a diagnosis from 1992 to 2006, 92.7% were automatched and a further 7.0% were matched manually, resulting in only 0.3% of the survivors missing a vital status. Finally, our study is limited by the fact that our death classification relied on the underlying cause of death as coded on the death certificate. Death certificates have been shown to be imperfect,^35^ ^36^ ^37^ ^38^ ^39^ and thus some degree of misclassification is inherent in our data. None the less, it is more likely that we have under-ascertained deaths due to non-neoplastic causes, which were largely the outcomes of interest in this study, as survivors of childhood cancer are more likely to be coded as having a neoplastic related death owing to their previous medical history.^20^ Thus, our results likely underestimated the risk of deaths due to non-neoplastic causes among survivors of childhood cancer, and so the real risks are likely to be even greater than stated in this report.

### Future research

As treatment regimens for childhood cancer are constantly evolving, reassessment of late effects in this population will be necessary. Although we observed significant declines in excess mortality among those with a more recent diagnosis, it will be important to assess with further follow-up whether the groups with most recent diagnoses remain at decreased risks. It is possible that death has only been delayed owing to increased awareness and surveillance of late effects, which could detect chronic health conditions earlier and in doing so potentially prolong life. 

This study also provides risk estimates among survivors of childhood cancer aged 60 years or more, where circulatory causes were found to be the main contributor to the excess number of deaths observed. Although we controlled for sex, type of first primary neoplasm, age at diagnosis, treatment period, and attained age, our results are only applicable to survivors who are at least 60 years old at the time of study, which is largely survivors of CNS neoplasms, Hodgkin’s lymphoma, and soft tissue sarcoma (see supplementary eTable 2). These survivors are quite different from those with a more recent diagnosis as they had a diagnosis of cancer that could be cured through surgery or radiotherapy alone (astrocytoma, CNS primitive neuroectodermal tumour—surgery and radiotherapy; early stage Hodgkin’s lymphoma—radiation; early stage soft tissue sarcoma—surgery and radiation). As the five year cure rates across most types of first primary neoplasm did not improve substantially until the 1970s, reassessment of the mortality risks in survivors beyond 60 years or more attained age will be necessary to determine the generalisability of our results. However, as circulatory causes are by far the leading cause of death in the general population for those living beyond 60 years of age, we suspect the findings presented in this study will also remain consistent for survivors with a more recent diagnosis.

### Conclusions and implications

The net effects of changes in cancer treatments, and surveillance and management for late effects, after treatment during the period 1940 to 2006 is to reduce the excess number of deaths from both recurrence or progression and non-neoplastic causes among those treated more recently. We provide evidence that among survivors aged at least 60 years, 31% and 37% of excess numbers of deaths observed were due to subsequent primary neoplasms and circulatory conditions, respectively. The fact that the excess numbers of deaths due to circulatory causes exceeds the excess number of deaths due to subsequent primary neoplasms is unsurprising because in the general population aged 60 years or more circulatory conditions account for substantially more deaths than neoplasms. The critically important message here for the evidence based surveillance aimed at preventing excess mortality and morbidity in survivors aged 60 years or more is that circulatory disease overtakes subsequent primary neoplasms as the leading cause of excess mortality; long term follow-up programmes must reflect this and target education, surveillance, and intervention appropriately.

**What is already known on this topic**

Survivors of childhood cancer are at an increased risk of death compared with the general population
The principal cause of excess mortality in the short term is recurrence or progression of the initial cancer, whereas subsequent primary neoplasms and non-neoplastic causes account for most excess deaths long term
Few previous studies have addressed late mortality in relation to treatment period, and those that have were restricted by narrow treatment periods, insufficient person years at risk, and small numbers of deaths, which limited statistical power and inhibited detailed classification and investigation of cause specific deaths

**What this study adds**

Among survivors of childhood cancer aged at least 60 years, 31% and 37% of excess numbers of deaths observed were due to subsequent primary neoplasms and circulatory conditions, respectively
The net effects of changes in cancer treatments, and surveillance and management for late effects, over the period 1940 to 2006 is to reduce the excess number of deaths from both recurrence or progression and non-neoplastic causes among those treated more recently

## Web extra

Extra material supplied by author

**Supplementary file:** eTables 1-3

## Notes

The British Childhood Cancer Survivor Study (BCCSS) is a national collaborative undertaking guided by a steering group that comprises Douglas Easton (chair), Michael Hawkins, Helen Jenkinson, Meriel Jenney, Raoul Reulen, Kathryn Pritchard-Jones, Michael Stevens, Elaine Sugden, Charles Stiller, Andrew Toogood, and Hamish Wallace. The BCCSS benefits from the contributions of the officers, centres, and individual members of the Children’s Cancer and Leukaemia Group and the Regional Paediatric Cancer Registries. The BCCSS acknowledges the collaboration of the Office for National Statistics, the National Records of Scotland, the Welsh Cancer Intelligence and Surveillance Unit, the Health & Social Care Information Centre, the National Cancer Intelligence Network, and Public Health England. The BCCSS would not have been possible without the support of our funders: Cancer Research UK and the European Commission to whom we offer our profound thanks. The views expressed in this publication are those of the authors and not necessarily represent those of the funders or collaborators. Finally, thanks to all BCCSS staff who have given many years of dedicated work to bring the BCCSS to fruition.

Ethical approval: This study was approved by the National Research Ethics Committee (10/H1102/86). The Confidentiality Advisory Group consented to processing identified data without individual patient consent (ECC 2-02 (f)/2011).

Data sharing: No additional data available.

Transparency: The lead author (MMF) and corresponding author (MMH) affirm that the manuscript is an honest, accurate, and transparent account of the study being reported, and that no aspects of the study have been omitted.
