# Supplementary material for: Long term cause specific mortality among 34 489 five year survivors of childhood cancer in Great Britain: population based cohort study
Source: BMJ. 2016 Sep 1;354:i4351. doi: 10.1136/bmj.i4351 (PMC5008696; doi:10.1136/bmj.i4351)
Supplement: Supplementary file 1 — Supplementary file: eTables 1-3 [file fidm032064.ww1_default.pdf]

Characteristic	Treatment Period							
	**1940-1969**	**%**	**1970-1979**	**%**	**1980-1989**	**%**	**1990-2006**	**%**
**Overall**	3746	10.9	5379	15.6	7147	20.7	18217	52.8
**Sex**								
Male	2019	53.9	2963	55.1	3941	55.1	10016	55.0
Female	1727	46.1	2416	44.9	3206	44.9	8201	45.0
**First Primary Neoplasm Type**								
CNS (excluding PNET)	1059	28.3	1094	20.3	1185	16.6	3632	19.9
CNS PNET	167	4.5	174	3.2	236	3.3	621	3.4
Leukemia (excluding AML)	168	4.5	1554	28.9	2206	30.9	5565	30.6
AML	10	0.3	68	1.3	193	2.7	710	3.9
Hodgkin Lymphoma	297	7.9	456	8.5	495	6.9	986	5.4
Non-Hodgkin Lymphoma	179	4.8	206	3.8	403	5.6	761	4.2
Neuroblastoma	217	5.8	153	2.8	307	4.3	858	4.7
Non-Heritable Retinoblastoma	255	6.8	195	3.6	160	2.2	396	2.2
Heritable Retinoblastoma	269	7.2	128	2.4	127	1.8	226	1.2
Wilms	324	8.7	480	8.9	515	7.2	1069	5.9
Bone Sarcoma	154	4.1	179	3.3	267	3.7	595	3.3
Soft Tissue Sarcoma	310	8.3	302	5.6	453	6.3	1082	5.9
Other	337	9.0	390	7.3	600	8.4	1716	9.4
**Age at Diagnosis**								
0-4	1727	46.1	2326	43.2	3317	46.4	8327	45.7
5-9	931	24.9	1567	29.1	1864	26.1	4902	26.9
10-14	1088	29.0	1486	27.6	1966	27.5	4988	27.4
**Follow-up (years)**								
Mean (SD)	41.3	16.9	33.3	11.0	26.7	6.6	14.7	5.0
5-9	458	12.2	524	9.7	459	6.4	3981	21.9
10-19	214	5.7	319	5.9	338	4.7	10750	59.0
20-29	166	4.4	243	4.5	4005	56.0	3486	19.1
30-39	202	5.4	2896	53.8	2345	32.8	0	0
40-49	1409	37.6	1397	26.0	0	0	0	0
50-59	1155	30.8	0	0	0	0	0	0
60+	142	3.8	0	0	0	0	0	0
**Attained Age (years)**								
Mean (SD)	47.8	16.9	40.0	11.5	33.3	7.9	21.3	6.5
5-9	86	2.3	105	2.0	95	1.3	565	3.1
10-19	405	10.8	478	8.9	429	6.0	7133	39,2
20-29	219	5.9	314	5.8	1474	20.6	8726	47.9
30-39	193	5.2	902	16.8	3730	52.2	1793	9.8
40-49	605	16.2	2826	52.5	1419	19.9	0	0
50-59	1343	35.9	754	14.0	0	0	0	0
60+	895	23.9	0	0	0	0	0	0

eTable 1: Cohort characteristics of the British Childhood Cancer Survivor Study by treatment period
Abbreviations: CNS – central nervous system, PNET – primitive neuroectodermal tumor, AML – acute myeloid leukemia

First Primary Neoplasm Type	Attained Age (years)													
	5-9	%	10-19	%	20-29	%	30-39	%	40-49	%	50-59	%	60+	%
CNS (excluding PNET)	159	18.7	1553	18.4	2258	21.0	1196	18.1	977	20.1	559	26.7	268	29.9
CNS PNET	19	2.2	371	4.4	381	3.6	193	2.9	148	3.1	62	3.0	24	2.3
Leukemia (excluding AML)	285	33.5	2987	35.4	3092	28.8	1892	28.6	1102	22.7	126	6.0	9	1.0
AML	36	4.2	320	3.8	363	3.4	181	2.7	58	1.2	22	1.1	1	0.1
Hodgkin Lymphoma	3	0.4	248	2.9	705	6.6	471	7.1	497	10.3	228	10.9	82	9.2
Non-Hodgkin Lymphoma	14	1.7	217	2.6	457	4.3	417	6.3	255	5.3	123	5.9	66	7.4
Neuroblastoma	100	11.8	550	6.5	412	3.8	225	3.4	135	2.8	95	4.5	18	2.0
Non-Heritable Retinoblastoma	31	3.6	214	2.5	230	2.1	154	2.3	201	4.1	122	5.8	54	6.0
Heritable Retinoblastoma	51	6.0	151	1.8	148	1.4	127	1.9	142	2.9	97	4.6	34	3.8
Wilms	48	5.6	624	7.4	627	5.8	488	7.4	405	8.4	155	7.4	41	4.6
Bone Sarcoma	4	0.5	182	2.2	365	3.4	270	4.1	203	4.2	100	4.8	71	7.9
Soft Tissue Sarcoma	35	4.1	454	5.4	676	6.3	409	6.2	306	6.3	173	8.3	94	10.5
Other	66	7.8	574	6.8	1019	9.5	595	9.0	421	8.7	235	11.2	133	14.9

eTable 2: First primary neoplasm types of the British Childhood Cancer Survivor Study by attained age

Abbreviations: CNS – central nervous system, PNET – primitive neuroectodermal tumor, AML – acute myeloid leukemia, SD – standard deviation

			All Causes			Recurrence/Progression		Subsequent Primary Neoplasms			Non-Neoplastic		
	**Treatment Period**	**Person-Years**	**O/E**	**SMR (95% CI)**	**AER (95% CI)**	**O/E**	**AER (95% CI)**	**O/E**	**SMR (95% CI)**	**AER (95% CI)**	**O/E**	**SMR (95% CI)**	**AER (95% CI)**
CNS (excluding PNET)	<1970	35,111	506/62.6	8.1 (7.4-8.8)	126.3 (113.7-138.8)	275/0.0	78.3 (69.1-87.6)	53/22.6	2.3 (1.8-3.1)	8.7 (4.6-12.7)	178/40.0	4.5 (3.8-5.2)	39.3 (31.9-46.8)
	1970-1979	30,505	335/27.2	12.3 (11.0-13.7)	100.9 (89.1-112.7)	193/0.0	63.3 (54.3-72.2)	41/6.2	6.6 (4.7-8.9)	11.4 (7.3-15.5)	101/20.9	4.8 (3.9-5.9)	26.2 (19.8-32.7)
	1980-1989	24,887	252/15.0	16.8 (14.8-19.0)	95.2 (82.7-107.7)	165/0.0	66.3 (56.2-76.4)	27/2.4	11.3 (7.5-16.5)	9.9 (5.8-14.0)	60/12.6	4.8 (3.6-6.1)	19.0 (12.9-25.1)
	1990-2006	34,248	241/10.8	22.3 (19.6-25.3)	67.2 (58.3-76.1)	175/0.0	51.1 (43.5-58.7)	27/1.5	18.3 (12.0-26.6)	7.5 (4.5-10.4)	39/9.3	4.2 (3.0-5.7)	8.7 (5.1-12.2)
P for trend				0.0397[b]	<0.0001[a]		<0.0001[a]		0.0006[b]	0.9094[a]		0.9024[b]	<0.0001[a]
CNS PNET	<1970	4,487	106/6.6	16.0 (13.1-19.3)	221.4 (176.5-266.4)	61/0.0	135.9 (101.8-170.1)	27/2.1	13.2 (8.7-19.2)	55.6 (32.9-78.3)	18/4.6	3.9 (2.3-6.2)	29.9 (11.4-48.4)
	1970-1979	4,160	76/3.5	21.9 (17.2-27.4)	174.3 (133.3-215.4)	39/0.0	93.8 (64.3-123.2)	18/0.7	26.5 (15.7-41.9)	41.6 (21.6-61.6)	19/2.8	6.8 (4.1-10.6)	39.0 (18.4-59.5)
	1980-1989	4,510	67/2.7	24.9 (19.3-31.7)	142.6 (107.0-178.2)	50/0.0	110.9 (80.1-141.6)	8/0.4	21.4 (9.2-42.1)	16.9 (4.6-29.2)	9/2.3	3.9 (1.8-7.4)	14.8 (1.8-27.9)
	1990-2006	5,538	91/1.8	50.4 (40.6-61.9)	161.1 (127.3-194.8)	65/0.0	117.4 (88.8-145.9)	19/0.2	80.9 (48.7-126.3)	33.9 (18.5-49.3)	7/1.6	4.5 (1.8-9.2)	9.8 (0.4-19.2)
P for trend				0.4198[b]	0.0207[a]		0.6074[a]		0.2312[b]	0.0710[a]		0.7987[b]	0.0436[a]
Leukemia (excluding AML)	<1970	3,256	98/4.1	24.0 (19.5-29.3)	288.4 (228.8-348.0)	94/0.0	288.7 (230.3-347.0)	1/1.3	0.8 (0.0-4.4)	-0.8 (-6.8-5.2)	3/2.8	1.1 (0.2-3.1)	0.6 (-9.9-11.0)
	1970-1979	40,452	414/29.2	14.2 (12.8-15.6)	95.1 (85.3-105.0)	309/0.0	76.4 (67.9-84.9)	50/5.9	8.4 (6.3-11.1)	10.9 (7.5-14.3)	55/23.3	2.4 (1.8-3.1)	7.8 (4.2-11.4)
	1980-1989	47,088	289/23.1	12.5 (11.1-14.1)	56.5 (49.4-63.6)	207/0.0	44.0 (38.0-49.9)	32/3.4	9.3 (6.4-13.1)	6.1 (3.7-8.4)	50/19.6	2.5 (1.9-3.4)	6.5 (3.5-9.4)
	1990-2006	54,441	303/14.8	20.4 (18.2-22.9)	52.9 (46.7-59.2)	220/0.0	40.4 (35.1-45.8)	43/2.1	20.6 (14.9-27.7)	7.5 (5.2-9.9)	40/12.7	3.1 (2.2-4.3)	5.0 (2.7-7.3)
P for trend				<0.0001[b]	<0.0001[a]		<0.0001[a]		0.1542[b]	0.3670[a]		0.9337[b]	0.3789[a]
AML	<1970	409	2/0.6	3.3 (0.4-11.9)	34.1 (-33.8-101.9)	1/0.0	24.5 (-23.5-72.5)	1/0.2	5.7 (0.1-31.7)	20.2 (-27.8-68.2)	0/0.4	0.0 (.-8.6)	-10.6 (-10.6--10.6)
	1970-1979	1,878	14/1.6	8.5 (4.7-14.3)	65.8 (26.8-104.9)	7/0.0	37.3 (9.7-64.9)	4/0.3	11.7 (3.2-30.1)	19.5 (-1.4-40.4)	3/1.3	2.3 (0.5-6.7)	9.0 (-9.0-27.1)
	1980-1989	3,976	36/2.0	18.1 (12.7-25.1)	85.6 (56.0-115.1)	15/0.0	37.7 (18.6-56.8)	7/0.3	22.5 (9.0-46.3)	16.8 (3.8-29.9)	14/1.7	8.4 (4.6-14.0)	31.0 (12.6-49.4)
	1990-2006	6,768	30/2.0	15.2 (10.3-21.7)	41.4 (25.6-57.3)	21/0.0	31.0 (17.8-44.3)	0/0.3	0	-0.4 (-0.4--0.4)	9/1.7	5.3 (2.4-10.1)	10.8 (2.1-19.5)
Ptrend				0.8310[b]	0.1324[a]		0.7522[a]		0.3467[b]	0.0071[a]		0.0225[b]	0.9294[a]
Hodgkin Lymphoma	<1970	8,690	156/19.2	8.1 (6.9-9.5)	157.4 (129.2-185.6)	87/0.0	100.1 (79.1-121.1)	33/6.5	5.1 (3.5-7.2)	30.5 (17.6-43.5)	36/12.8	2.8 (2.0-3.9)	26.7 (13.2-40.3)
	1970-1979	13,011	105/14.8	7.1 (5.8-8.6)	69.3 (53.9-84.8)	40/0.0	30.7 (21.2-40.3)	32/3.2	10.1 (6.9-14.3)	22.2 (13.6-30.7)	33/11.6	2.8 (2.0-4.0)	16.4 (7.8-25.1)
	1980-1989	11,424	42/9.1	4.6 (3.3-6.2)	28.8 (17.7-39.9)	19/0.0	16.6 (9.2-24.1)	10/1.4	7.1 (3.4-13.1)	7.5 (2.1-12.9)	13/7.7	1.7 (0.9-2.9)	4.6 (-1.6-10.8)
	1990-2006	9,475	28/4.2	6.6 (4.4-9.6)	25.1 (14.1-36.0)	14/0.0	14.8 (7.0-22.5)	5/0.5	9.8 (3.2-22.8)	4.7 (0.1-9.4)	9/3.7	2.4 (1.1-4.6)	5.6 (-0.6-11.8)
P for trend				<0.0001[b]	<0.0001[a]		<0.0001[a]		0.7078[b]	0.0002[a]		0.1414[b]	0.0026[a]
Non-Hodgkin Lymphoma	<1970	7,071	46/15.7	2.9 (2.1-3.9)	42.8 (24.0-61.6)	12/0.0	17.0 (7.4-26.6)	12/5.6	2.2 (1.1-3.8)	9.1 (-0.5-18.7)	22/10.2	2.2 (1.4-3.3)	16.7 (3.7-29.7)
	1970-1979	6,264	27/6.5	4. 2 (2.7-6.1)	32.8 (16.5-49.0)	11/0.0	17.6 (7.2-27.9)	9/1.3	6.8 (3.1-12.8)	12.2 (2.9-21.6)	7/5.2	1.4 (0.5-2.8)	2.9 (-5.3-11.2)
	1980-1989	9,047	31/6.3	4.9 (3.3-6.9)	27.3 (15.2-39.3)	11/0.0	12.2 (5.0-19.3)	5/0.9	5.7 (1.8-13.3)	4.6 (-0.3-9.4)	15/5.5	2.7 (1.5-4.5)	10.5 (2.2-18.9)
	1990-2006	7,962	27/3.3	8.1 (5.3-11.8)	29.7 (16.9-42.5)	14/0.0	17.6 (8.4-26.8)	5/0.4	12.5 (4.1-29.2)	5.8 (0.3-11.3)	8/2.9	2.7 (1.2-5.4)	6.4 (-0.6-13.3)
P for trend				0.9035[b]	0.2571[a]		0.8643[a]		0.3087[b]	0.8793[a]		0.9388[b]	0.3995[a]

eTable 3: Standardized mortality ratios and absolute excess risks for all, recurrence or progression, subsequent primary neoplasm, and non-neoplastic causes-of-death, by first primary neoplastic type and treatment period

Abbreviations: CNS – central nervous system, PNET – primitive nervous system tumor, AML – acute myeloid leukemia, O – observed, E – expected, SMR – standardized mortality ratio, AER – absolute excess risk, CI – confidence intervals, NA – not applicable, NP -not possible to reliably calculate due to very small numbers

[a]P for trend determined from univariable Poisson model

[b]P for trend determined from multivariable Poisson model adjusting for sex, first primary neoplasm type, age at diagnosis, treatment period, and attained age

			All Causes			Recurrence/Progression		Subsequent Primary Neoplasms			Non-Neoplastic		
	Treatment Period	Person-Years	O/E	SMR (95%CI)	AER (95%CI)	O/E	AER (95%CI)	O/E	SMR (95%CI)	AER (95%CI)	O/E	SMR (95%CI)	AER (95%CI)
Neuroblastoma	<1970	8,798	49/10.1	4.8<br>(3.6-6.4)	44.2<br>(28.6-59.8)	18/0.0	20.5<br>(11.0-29.9)	11/3.1	3.6<br>(1.8-6.4)	9.0<br>(1.6-16.4)	20/7.1	2.8<br>(1.7-4.4)	14.7<br>(4.7-24.7)
	1970-1979	4,584	23/2.7	8.4<br>(5.3-12.6)	44.2<br>(23.7-64.7)	13/0.0	28.4<br>(12.9-43.8)	5/0.5	10.7<br>(3.5-24.9)	9.9<br>(0.3-19.4)	5/2.3	2.2<br>(0.7-5.2)	6.0<br>(-3.6-15.5)
	1980-1989	6,739	29/2.5	11.6<br>(7.8-16.7)	39.3<br>(23.7-55.0)	17/0.0	25.2<br>(13.2-37.2)	4/0.4	11.2<br>(3.1-28.7)	5.4<br>(-0.4-11.2)	8/2.1	3.7<br>(1.6-7.4)	8.7<br>(0.5-16.9)
	1990-2006	8,380	43/1.5	28.9<br>(20.9-38.9)	49.5<br>(34.2-64.9)	37/0.0	44.2<br>(29.9-58.4)	2/0.3	7.7<br>(0.9-27.9)	2.1<br>(-1.2-5.4)	4/1.2	3.3<br>(0.9-8.3)	3.3<br>(-1.4-8.0)
P for trend				0.0212[b]	0.7274[a]		0.0082[a]		0.6128[b]	0.0842[a]		0.9201	0.0596[a]
Non-heritable Retinoblastoma	<1970	11,822	19/17.0	1.1<br>(0.7-1.8)	1.7<br>(-5.5-9.0)	0/0.0	0	13/5.8	2.2<br>(1.2-3.8)	6.1<br>(0.1-12.1)	6/11.2	0.5<br>(0.2-1.2)	-4.4<br>(-8.4--0.3)
	1970-1979	6,498	9/4.1	2.2<br>(1.0-4.2)	7.6<br>(-1.5-16.6)	0/0.0	0	1/0.7	1.4<br>(0.0-7.7)	0.4<br>(-2.6-3.4)	8/3.4	2.4<br>(1.0-4.7)	7.1<br>(-1.4-15.7)
	1980-1989	3,720	2/1.4	1.4<br>(0.2-5.2)	1.6<br>(-5.8-9.1)	0/0.0	0	1/0.2	5.1<br>(0.1-28.3)	2.2<br>(-3.1-7.4)	1/1.2	0.8<br>(0.0-4.7)	-0.5<br>(-5.8-4.8)
	1990-2006	4,127	1/0.8	1.3<br>(0.0-7.3)	0.6<br>(-4.2-5.3)	0/0.0	0	0/0.1	0	-0.3<br>(-0.3--0.3)	1/0.6	1.6<br>(0.0-8.8)	0.9<br>(-3.9-5.6)
P for trend				0.5205[b]	0.9396[a]		*NA*		0.6345[b]	0.1790[a]		0.2701[b]	0.5799[a]
Heritable Retinoblastoma	<1970	10,939	96/13.2	7.3<br>(5.9-8.9)	75.7<br>(58.1-93.2)	11/0.0	10.1<br>(4.1-16.0)	71/4.1	17.2<br>(13.4-21.7)	61.1<br>(46.0-76.2)	14/9.1	1.5<br>(0.8-2.6)	4.5<br>(-2.2-11.2)
	1970-1979	3,996	21/2.3	9.2<br>(5.7-14.1)	46.8<br>(24.4-69.3)	3/0.0	7.5<br>(-1.0-16.0)	15/0.4	39.1<br>(21.9-64.4)	36.6<br>(17.6-55.6)	3/1.9	1.6<br>(0.3-4.6)	2.8<br>(-5.7-11.3)
	1980-1989	2,835	17/1.0	17.0<br>(9.9-27.3)	56.4<br>(27.9-84.9)	5/0.0	17.6<br>(2.2-33.1)	11/0.1	77.6<br>(38.7-138.9)	38.3<br>(15.4-61.2)	1/0.9	1.2<br>(0.0-6.5)	0.5<br>(-6.4-7.4)
	1990-2006	2,392	4/0.4	9.9<br>(2.7-25.4)	15.0<br>(-1.4-31.4)	2/0.0	8.4<br>(-3.2-20.0)	2/0.1	27.4<br>(3.3-99.0)	8.1<br>(-3.5-19.6)	0/0.3	0	-1.4<br>(-1.4--1.4)
P for trend				0.6438[b]	0.0014[a]		0.7669[a]		0.7772[b]	0.0004[a]		0.6380[b]	0.2283[a]
Wilms	<1970	13,514	75/16.6	4.5<br>(3.6-5.7)	43.2<br>(30.7-55.8)	11/0.0	8.1<br>(3.3-12.9)	29/5.2	5.6<br>(3.7-8.0)	17.6<br>(9.8-25.4)	35/11.4	3.1<br>(2.1-4.3)	17.5<br>(8.9-26.1)
	1970-1979	15,121	65/10.0	6.5<br>(5.0-8.3)	36.3<br>(25.9-46.8)	19/0.0	12.6<br>(6.9-18.2)	17/1.8	9.4<br>(5.4-15.0)	10.0<br>(4.7-15.4)	29/8.2	3.5<br>(2.4-5.1)	13.7<br>(6.8-20.7)
	1980-1989	11,964	29/4.9	5.9<br>(3.9-8.4)	20.1<br>(11.3-28.9)	10/0.0	8.4<br>(3.2-13.5)	3/0.7	4.2<br>(0.9-12.3)	1.9<br>(-0.9-4.7)	16/4.2	3.8<br>(2.2-6.2)	9.8<br>(3.3-16.4)
	1990-2006	10,920	15/2.3	6.6<br>(3.7-10.8)	11.6<br>(4.7-18.6)	12/0.0	11.0<br>(4.8-17.2)	0/0.4	0	-0.3<br>(-0.3--0.3)	3/1.9	1.6<br>(0.3-4.6)	1.0<br>(-2.1-4.1)
P for trend				0.8409[b]	<0.0001[a]		0.7485[a]		0.1683[b]	<0.0001[a]		0.2889[b]	0.0117[a]
Bone Sarcoma	<1970	5,634	48/13.2	3.6<br>(2.7-4.8)	61.9<br>(37.8-86.0)	25/0.0	44.4<br>(27.0-61.8)	13/5.2	2.5<br>(1.3-4.3)	13.9<br>(1.4-26.5)	10/8.0	1.3<br>(0.6-2.3)	3.6<br>(-7.4-14.6)
	1970-1979	5,004	41/5.3	7.8<br>(5.6-10.5)	71.4<br>(46.3-96.4)	23/0.0	46.0<br>(27.2-64.7)	10/1.3	7.6<br>(3.6-13.9)	17.3<br>(5.0-29.7)	8/4.0	2.0<br>(0.9-4.0)	8.1<br>(-3.0-19.1)
	1980-1989	5,635	44/3.9	11.3<br>(8.2-15.1)	71.2<br>(48.1-94.2)	29/0.0	51.5<br>(32.7-70.2)	11/0.7	16.6<br>(8.3-29.7)	18.3<br>(6.8-29.9)	4/3.2	1.2<br>(0.3-3.2)	1.4<br>(-5.6-8.3)
	1990-2006	5,524	65/2.3	28.5<br>(22.0-36.3)	113.5<br>(84.9-142.1)	58/0.0	105.0<br>(78.0-132.0)	3/0.3	10.2<br>(2.1-29.9)	4.9<br>(-1.2-11.0)	4/2.0	2.0<br>(0.5-5.2)	3.6<br>(-3.5-10.7)
P for trend				0.0728[b]	0.0110[a]		0.0001[a]		0.5865[b]	0.2928[a]		0.8644[b]	0.6998[a]
Soft Tissue Sarcoma	<1970	12,458	60/22.9	2.6<br>(2.0-3.4)	29.8<br>(17.6-42.0)	26/0.0	20.9<br>(12.8-28.9)	13/8.0	1.6<br>(0.9-2.8)	4.0<br>(-1.7-9.7)	21/14.8	1.4<br>(0.9-2.2)	5.0<br>(-2.3-12.2)
	1970-1979	8,739	66/7.3	9.0<br>(7.0-11.4)	67.1<br>(48.9-85.3)	34/0.0	38.9<br>(25.8-52.0)	17/1.5	11.1<br>(6.4-17.7)	17.7<br>(8.4-26.9)	15/5.8	2.6<br>(1.4-4.3)	10.5<br>(1.8-19.2)
	1980-1989	9,975	53/5.6	9.5<br>(7.1-12.4)	47.5<br>(33.2-61.8)	35/0.0	35.1<br>(23.5-46.7)	9/0.9	10.5<br>(4.8-20.0)	8.2<br>(2.3-14.1)	9/4.7	1.9<br>(0.9-3.6)	4.3<br>(-1.6-10.2)
	1990-2006	10,891	74/3.4	21.6<br>(16.9-27.1)	64.8<br>(49.3-80.3)	54/0.0	49.6<br>(36.4-62.8)	11/0.5	23.5<br>(11.7-42.0)	9.7<br>(3.7-15.6)	9/3.0	3.0<br>(1.4-5.8)	5.5<br>(0.1-10.9)
P for trend				0.0032[b]	0.0140[a]		0.0006[a]		0.0004[b]	0.3869[a]		0.6827[b]	0.9485[a]
Other	<1970	13,695	68/26.3	2.6<br>(2.0-3.3)	30.4<br>(18.6-42.2)	17/0.0	12.4<br>(6.5-18.3)	18/10.1	1.8<br>(1.1-2.8)	5.7<br>(-0.3-11.8)	33/16.2	2.0<br>(1.4-2.9)	12.3<br>(4.1-20.5)
	1970-1979	12,067	51/10.5	4.9<br>(3.6-6.4)	33.6<br>(22.0-45.2)	17/0.0	14.1<br>(7.4-20.8)	21/2.9	7.2<br>(4.4-11.0)	15.0<br>(7.5-22.4)	13/7.5	1.7<br>(0.9-3.0)	4.5<br>(-1.3-10.4)
	1980-1989	13,576	51/7.5	6.8<br>(5.1-9.0)	32.1<br>(21.7-42.4)	28/0.0	20.6<br>(13.0-28.3)	10/1.4	7.4<br>(3.5-13.6)	6.4<br>(1.8-10.9)	13/6.1	2.1<br>(1.1-3.6)	5.1<br>(-0.1-10.3)
	1990-2006	16,552	35/5.3	6.6<br>(4.6-9.2)	18.0<br>(11.0-25.0)	17/0.0	10.3<br>(5.4-15.2)	5/0.7	6.7<br>(2.2-15.6)	2.6<br>(-0.1-5.2)	13/4.5	2.9<br>(1.5-4.9)	5.1<br>(0.9-9.4)
P for trend				0.2234[b]	0.0115[a]		0.8740[a]		0.6745[b]	0.0187[a]		0.7290[b]	0.0947[a]

eTable 3 (*continued*): Standardized mortality ratios and absolute excess risks for all, recurrence or progression, subsequent primary neoplasm, and non-neoplastic causes-of-death, by first primary neoplastic type and treatment period

Abbreviations: CNS – central nervous system, PNET – primitive nervous system tumor, AML – acute myeloid leukemia, O – observed, E – expected, SMR – standardized mortality ratio, AER – absolute excess risk, CI – confidence intervals, NA – not applicable, NP -not possible to reliably calculate due to very small numbers

[a]P for trend determined from univariable Poisson model

[b]P for trend determined from multivariable Poisson model adjusting for sex, first primary neoplasm type, age at diagnosis, treatment period, and attained age
